# A Joint Analysis of RNA-DNA and DNA-DNA Interactomes Reveals Their Strong Association

**DOI:** 10.3390/ijms26031137

**Published:** 2025-01-28

**Authors:** Dmitry S. Zvezdin, Artyom A. Tyukaev, Anastasia A. Zharikova, Andrey A. Mironov

**Affiliations:** 1 RSC Bioinformatics, Kharkevich Institute for Information Transmission Problems, Russian Academy of Sciences, Bolshoy Karetny Per. 19, 127051 Moscow, Russia; azharikova89@gmail.com; 2 Faculty of Bioengineering and Bioinformatics, Lomonosov Moscow State University, 1-7-3 Leninskie Gory, 119991 Moscow, Russia; artyomtyukaev@gmail.com; 3 Vavilov Institute of General Genetics, Russian Academy of Sciences, Gubkina Str. 3, 119333 Moscow, Russia; 4 National Medical Research Center for Therapy and Preventive Medicine, Ministry of Healthcare of the Russian Federation, Petroverigsky Per. 10, 101000 Moscow, Russia

**Keywords:** RNA-DNA interactome, non-coding RNAs, chromatin, Hi-C

## Abstract

At the moment, many non-coding RNAs that perform a variety of functions in the regulation of chromatin processes are known. An increasing number of protocols allow researchers to study RNA-DNA interactions and shed light on new aspects of the RNA–chromatin interactome. The Hi-C protocol, which enables the study of chromatin’s three-dimensional organization, has already led to numerous discoveries in the field of genome 3D organization. We conducted a comprehensive joint analysis of the RNA-DNA interactome and chromatin structure across different human and mouse cell lines. We show that these two phenomena are closely related in many respects, with the nature of this relationship being both tissue specific and conserved across humans and mice.

## 1. Introduction

Most of the human genome is transcribed, and the proportion of protein-coding genes is small among these transcripts [1]. The vast majority of transcribed loci are represented by various classes of non-coding RNAs that perform a variety of functions in the nuclei of eukaryotic cells. Small nuclear RNAs are key factors in splicing, small nucleolar RNAs are involved in the processing of ribosomal RNAs, and long non-coding RNAs play diverse roles in chromatin regulation [2]. MALAT1 participates in the organization of nuclear speckles [3], NEAT1 is a key structural element of paraspeckles [4], XIST participates in dose compensation of the second X chromosome of female mammals [5], and FIRRE plays a role in mediating interchromosomal contacts through proteins, thus helping to maintain chromatin structure [6]. In addition, several proteins that play a key role in maintaining the spatial structure of chromatin, such as CTCF and components of the PRC2 protein complex, have the ability to bind RNA, which indicates the possibility of involving RNA molecules in the regulatory function of these proteins [2].

In recent years, a number of methods have been developed to map the RNA–chromatin interactions. First, the methods from the “one-to-all” group appeared (RAP [7], CHIRP [8], CHART [9]), which allow us to study the interactions of one selected RNA with the whole genome with good coverage. To be able to map the interactions of all RNAs genome-wide, methods from the “all-to-all” group were developed (Red-C [10], GRID-seq [11], RADICL-seq [12], MARGI [13], iMARGI [14]). In addition to the obvious advantages, they also have a number of disadvantages, namely, a high level of noise and very significant incompleteness of data. Despite these limitations, “all-to-all” methods have provided new insights into the RNA-DNA interactome. For example, in a study using the Red-C method, the authors annotated a variety of previously unannotated chromatin-associated RNAs (ucaRNA) which had not been detected before, likely due to their strong tendency to localize on chromatin [10]. Data for the RNA–chromatin interactome have a number of biases, in particular, a decrease in the density of contacts with an increase in the distance from the gene encoding the RNA. This shift in the data is caused by contacts of the transcribed RNA (polymerase trace) and the diffusion of RNA through the nucleus. In the future, by analogy with the Hi-C data, we will call this effect RD-scaling.

The Hi-C experimental protocol produces genome-wide mapping of DNA-DNA interactions [15]. Using these data, many discoveries have been made in recent years in the study of the structural organization of chromatin. Various spatial structures formed by chromosomes have been annotated on Hi-C maps, such as topologically associated domains (TADs) [16], chromatin loops [17], and A/B compartments [15]. Several studies have examined the relationship between RNA-DNA interactions and chromatin structures, including TADs and A/B compartments. It was found that contacts of most RNAs tend to be localized within the TAD of their gene [11,12]. A preference for interacting with loci in the compartment containing the RNA gene was also observed [12]. However, these analyses were mostly general, and thorough study at the level of individual RNAs was not performed. A large-scale study of the relationship of RNA-DNA interactions and the chromatin structure for K562 cells was conducted in 2023 [18]. The barrier effect of the TAD boundaries was experimentally confirmed, and it was also shown that inhibition of transcription and treatment with RNase lead to the formation of new chromatin loops.

In a 2024 study [19], it was shown using the data of the iMARGI protocol that RNA-DNA interactions improve the quality of prediction of Hi-C maps based on the DNA sequence with machine learning models. The HiChIRP method was also developed, combining the study of the RNA-DNA interactome and the spatial structure of chromatin [20]. It allows researchers to study DNA-DNA interactions associated with a specific RNA, but it involves the mapping of only a limited set of predefined RNAs. Attempts to perform a genome-wide characterization of the RNAs involved in regulatory processes in chromatin based on a joint analysis of the RNA-DNA interactome and chromatin structure have not yet been made.

**Figure 1:**
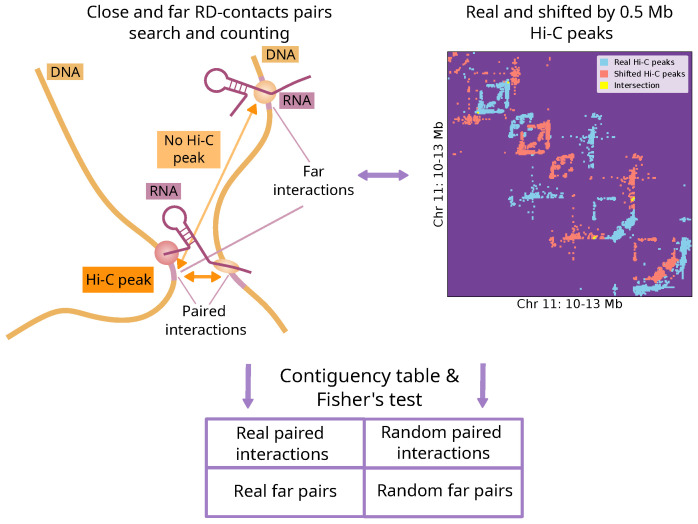
The analysis scheme of the association of chromatin structure and contacts of individual RNAs. Structurally associated contacts are defined based on the Hi-C map. A random model is constructed by shifting the Hi-C map. The statistical significance for the association with chromatin structure is tested for each RNA on each chromosome.

In this paper, we propose an approach for the joint analysis of the RNA-DNA interactome and chromatin structure (Figure 1). We show using a number of cell lines that the distribution of RNA contacts across the genome is related to its spatial organization at the intrachromosomal and interchromosomal levels. We demonstrate functional differences in RNA-associated chromatin structures for different RNAs and highlight the conservation of the RNA-DNA interactome and chromatin structure association across experiments. We also conduct a comprehensive analysis of the relationship between RNA-DNA interaction patterns and certain chromatin structures. Our study shows that many RNAs tend to interact with chromatin loops. We demonstrate that RNAs preferentially interact with the boundaries of their TADs, and the tendency of RNAs to localize outside their parental TAD is partially conserved across humans and mice and is dependent on the RNA biotype. The entire analysis is performed using the BaRDIC peak caller for RNA-DNA interactions, taking into account the effects of the background and RD-scaling [21]. In this study, we adopt the terminology commonly used in studies of chromatin structure; we define cis-contacts as interactions with the chromosome on which the RNA gene is located and trans-contacts as interactions with other chromosomes.

## 2. Results

### 2.1. The Contacts of Many RNAs Are Associated with the Chromatin Structure

One of the main goals of this work was to determine whether there is an association between the distribution of individual RNA contacts across the genome and its three-dimensional structure. To answer this question, we proposed the procedure which is described in detail in the methods. We considered all possible pairs of contacts between certain RNAs and chromatin. For each pair, we asked whether the contacts were spatially close, as defined by a Hi-C map (hereinafter referred to as paired contacts) (Figure 1). To assess the statistical significance of the observations, we used a background model by shifting the coordinates of Hi-C interactions (example in Figure S1). The procedure was performed on eight “all-to-all” datasets (datasets have different quality and coverage, detailed characteristics are provided in Table S1). For each dataset, we selected the 6000 RNAs with the largest number of peaks from the BaRDIC tool and Hi-C peaks with an FDR (FitHiC2 method) of 0.05. We call Hi-C significant interactions Hi-C peaks by analogy with ChIP-seq data.

**Figure 2:**
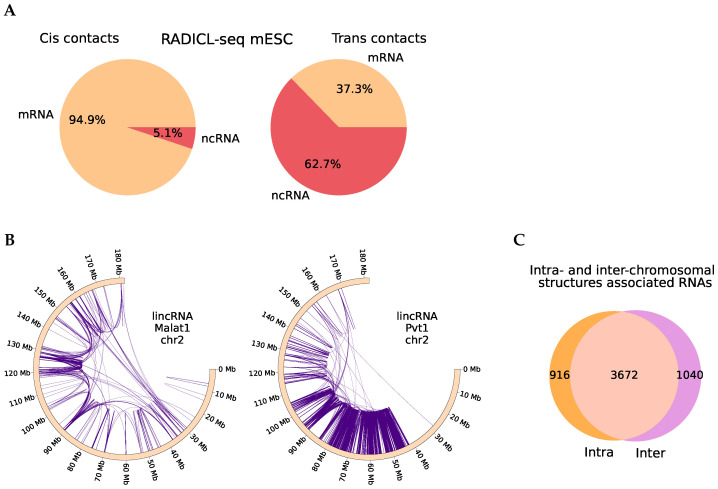
(**A**) The distribution of significant results by RNA types. On the **left**—on RNAs’ gene chromosome; on the **right**—on the other chromosomes (mESC RADICL-seq). (**B**) Examples of paired contacts of long non-coding RNA of mOPC RADICL-seq data: Malat1 (chr2) on the left, Pvt1 (chr2) on the right. (**C**) Intersections of RNAs significantly associated with the chromatin structure in the interchromosomal and intrachromosomal cases (mESC RADICL-seq).

First, we analyzed the association of the RNA contacts on each chromosome with intrachromosomal Hi-C maps. For the RADICL-seq data for the mouse embryonic stem cells (mESC) cell line, we identified 4588 RNAs whose contacts were significantly associated with chromatin structures on at least one chromosome. On the chromosome containing the RNA gene, the number of significant results (at an FDR level < 0.05) corresponding to mRNAs was approximately equal to the background number of mRNAs in the data (92.4%). On other chromosomes, results related to non-coding RNAs were overrepresented (Figure 2A). This suggests that the chromatin structure plays a role in localization around the RNA gene, regardless of its biotype. On other chromosomes, it is mainly associated with contacts of non-coding RNAs, which are often involved in regulatory roles. We obtained a consistent distribution of results from other experiments (statistics for all protocols are shown in Figure S2). Figure 2B shows the paired contacts of long non-coding RNAs (mouse oligodendrocytes progenitor cells (mOPC) RADICL-seq) Malat1 and Pvt1 on chromosome 2. The distribution of the paired contacts differs. Malat1 tends to interact along distant chromosomal structures, which likely represent distant interactions between the active chromatin compartments. Pvt1 interacts with more local structures (other examples are given in Figures S3 and S4).

Similarly, we analyzed the association of RNA-DNA interactions and chromatin structure at the interchromosomal level using FitHiC2 peaks on interchromosomal Hi-C maps with an FDR of 0.2. For each RNA, we considered all possible pairs of contacts between each pair of chromosomes and performed a similar calculation and statistical analysis using Hi-C interchromosomal maps. We also obtained significant results for many RNAs in both mouse (for 4712/6000 RNAs based on mESC RADICL-seq data) and human cells. Figure S5 shows the paired contacts for lncRNA Malat1 (mESC RADICL-seq) on the left. These are not separate pairs of contacts but a “network” of many loci, which corresponds to the results of the RD-SPRITE method [22]. Malat1 is involved in the formation of speckles [3], and regions associated with speckles have been shown to exhibit a tendency for interchromosomal interactions [23], which is consistent with the results of this analysis. Figure 2C shows the intersections of RNAs whose contacts are associated with the chromatin structure at the intrachromosomal and interchromosomal levels. Although most RNAs showed significant results in both cases, we found examples with significant paired contacts only between different chromosomes (Table S2). This may be explained by their predominant localization at the borders of chromosomal territories. Figure S5 shows an example of the interchromosomal paired contacts of such an RNA on the right. K562 cells are characterized by chromosomal rearrangements, so we did not interpret their RNAs’ interchromosomal paired contacts (details in Supplementary Note S1 and Figure S6).

“All-to-all” datasets have different coverage, quality, and technical features. To ensure that the obtained results were not critically influenced by these factors, we analyzed data from “one-to-all” protocols for several RNAs (listed in Table S3 from the mESC cell line. We also observed the structural association of contacts in all experiments at both the intrachromosomal and interchromosomal levels. For example, in one of the experiments for Malat1, we observed a significant enrichment of paired contacts on all chromosomes and between all chromosome pairs. Thus, with sufficient coverage, the observed relationship becomes even more pronounced.

### 2.2. Structurally Associated Contacts Are Connected with Functional Chromatin Annotations

**Figure 3:**
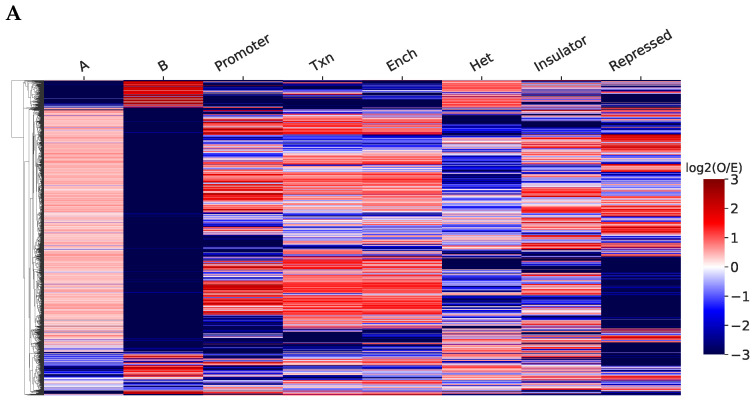

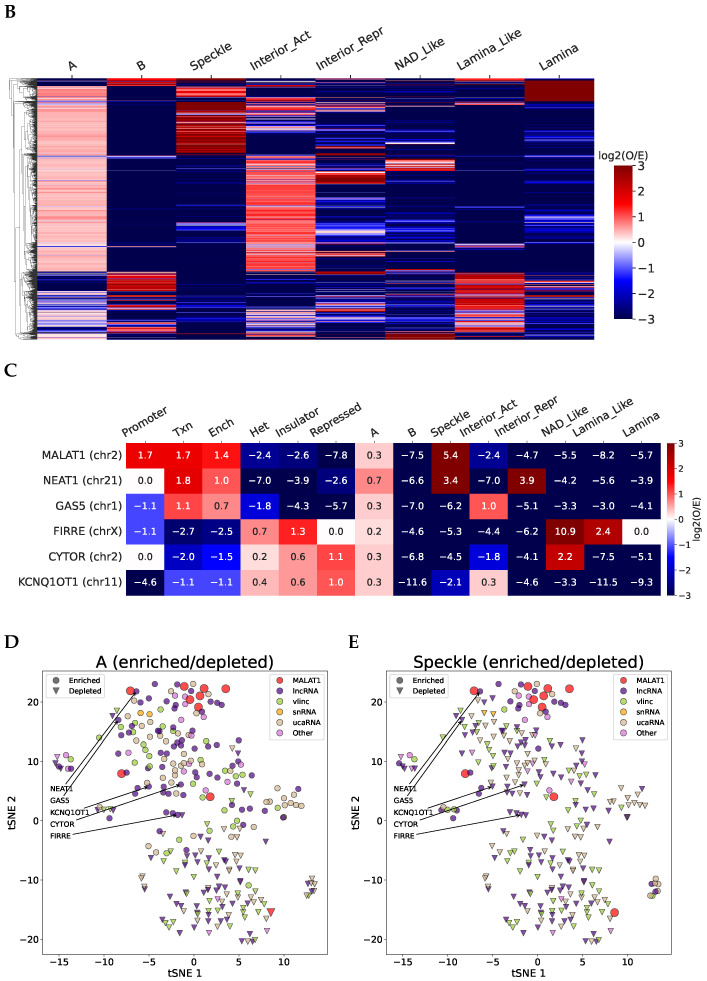
(**A**,**B**) Heat maps of the representation of the observed paired contacts in the states of chromatin markings relative to the expected value ((**A**) A/B compartments + chromHMM, (**B**) A/B compartments + SPIN states). (**C**) Heat maps for known long non-coding RNAs. Log2 values of observed-to-expected ratios of paired interactions in chromatin functional annotation states are shown. (**D**,**E**) Visualization of non-coding RNAs in the tSNE space based on log2 observed/expected paired contacts in all considered states. Markers show enrichment/depletion of paired contacts in states pairs, colors show RNAs types ((**D**) A compartment, (**E**) speckles).

Next, we questioned whether we can characterize the functional role of the defined RNAs through the annotation of chromatin interactions. For K562 cells, we annotated Hi-C interactions with pairs of chromatin functional states, namely, ChromHMM and SPIN, as well as A/B compartments. We annotated the intrachromosomal paired contacts of RNAs associated with chromatin structure and analyzed their representation in pairs of chromatin states, relative to the total number of Hi-C interactions within these states. We analyzed only pairs of states with the same name (Figure 3A,B), since loci belonging to different states interact much less intensively with each other [24,25]. For ChromHMM, in general, paired contacts are overrepresented in active chromatin (enhancers, promoters, transcription). However, there are also many cases of overrepresentation in insulators and repressed chromatin, which may be due to the involvement of a number of RNAs in the regulation of CTCF and the PRC2 complex. Paired contacts are poorly represented in heterochromatin, which is likely due to the reduced density of observed RNA contacts with inactive chromatin. For SPIN states, which characterize the association of chromatin sites with nuclear structures (Figure 3B), the propensity of individual RNAs to interact with certain states was more pronounced than in a similar plot for ChromHMM. This is likely because the authors of SPIN took into account the spatial proximity of the states of the same name based on Hi-C data [26], which aligns with our paired contacts analysis. Within the compartments, paired contacts of most of the RNAs prevail in the active chromatin of the A compartment.

For a comprehensive characterization of RNA functions, we combined data on all three annotations and analyzed individual long non-coding RNAs with known functions (Figure 3C). The results we observed were mostly consistent with those expected based on the literature. MALAT1 prefers to interact within the active chromatin states (ChromHMM: promoters, enhancers, transcription) on almost all chromosomes on which paired contacts were found. In SPIN states, its paired contacts are overrepresented in speckles, which is consistent with its function in their organization (Figure S7). The behavior of NEAT1 is generally similar to MALAT1, which is consistent with the data supporting their colocalization [27]. For the GAS5 lncRNA, we primarily assumed an association with active chromatin, based on the literature [28]. We observed a similar pattern for GAS5; it preferably contacts within the active chromatin and avoids interacting with the structures of repressed chromatin and heterochromatin. As expected, paired contacts of the KCNQ1OT1 lncRNA, which is involved in imprinting the nearby locus [29], are enriched in repressed chromatin and depleted in active chromatin states. The association of FIRRE RNA with CTCF has been previously shown [6], and we observed this in the enrichment of its paired contacts in pairs of insulators. Additionally, FIRRE showed overrepresented paired contacts in nucleolus-associated regions, which is consistent with the established participation of FIRRE in the colocalization of the inactivated X chromosome with the nucleolus in the mouse cells [30]. CYTOR is known to interact with the nucleolar protein nucleolin [31], and its paired contacts are overrepresented in nucleolus-associated sites. CYTOR and KCNQ1OT1 are also overrepresented simultaneously in active A-compartment and PRC-repressed chromatin. This is possibly due to most of the ChromHMM repressed chromatin intersecting with the A compartment (87.2%). This observation is consistent with other works showing the presence of H3K27me3 histone modification not only in the inactive B compartment but in the A compartment too [15,17]. Some other inconsistent results are described in Supplementary Note S2.

We used the combined annotation to analyze the functions of other non-coding RNAs. Figure 3D,E show RNAs in t-SNE coordinates based on the observed-to-expected ratio of all the above-mentioned annotations. RNAs are colored by biotypes, and the marker indicates cases of enrichment and depletion of paired contacts in the A compartment and speckles (other groups are shown in Figure S8). It can be seen that RNAs are divided into several groups, NEAT1, GAS5, and MALAT1, and small nuclear RNAs form a group that prefers active chromatin. KCNQ1OT1 and CYTOR are grouped based on the enrichment of paired contacts in repressed chromatin. There is a clear division of RNA into groups according to compartment preference. A group of RNAs that prefer to contact speckles is separated, and MALAT1 on different chromosomes and NEAT1 form a significant part of this group.

For most of the considered RNAs with known functions, we obtained results consistent with the literature. Then, we analyzed less or completely unexplored but highly contacting RNAs (details are in Supplementary Note S2 and Supplementary Data).

### 2.3. The Association of the RNA-DNA Interactome and Chromatin Structure Is Conserved Across Experiments

**Figure 4:**
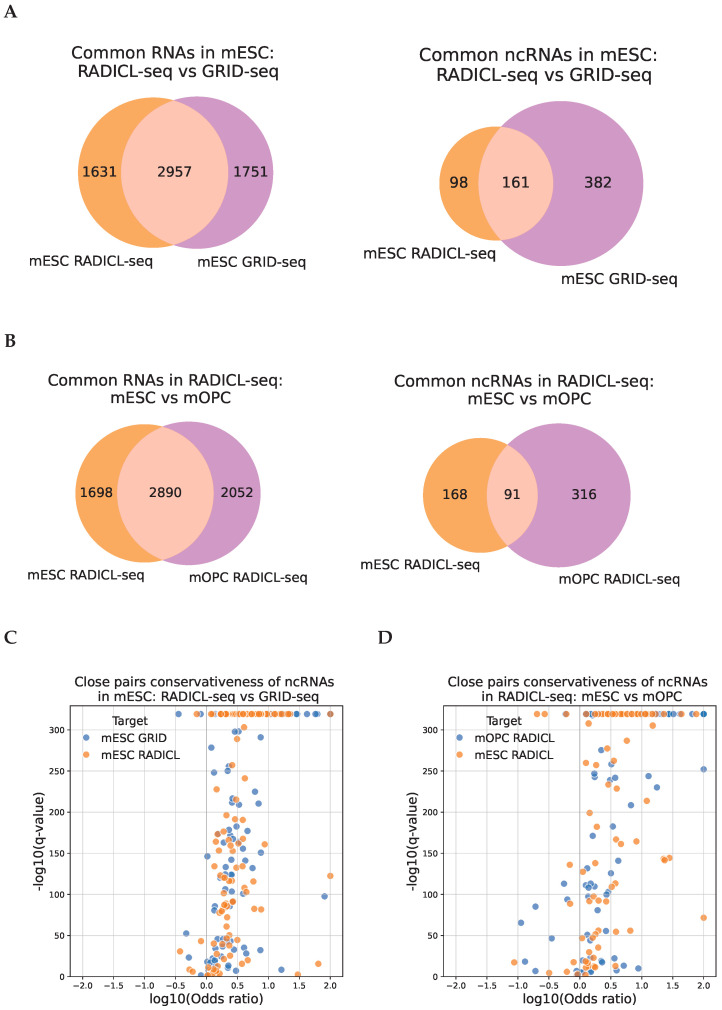
(**A**,**B**) Intersection of sets of RNAs associated with the chromatin structure in mice: on the **left**—all RNAs; on the **right**—ncRNAs. ((**A**) between mESC cells based on the RADICL-seq and GRID-seq protocols, (**B**) between mESC and mOPC cells of the RADICL-seq protocol). (**C**,**D**) The scatterplot of the q-values and the odds ratios of the Fisher’s exact test for paired interactions conservation between protocols and cell lines. ((**C**) Comparison of GRID-seq and RADICL-seq protocols, (**D**) comparison of mESC and mOPC cell lines).

It is known from the literature that the three-dimensional structure of chromatin is partially conserved across cell lines during differentiation [32]. We questioned the conservation of the RNA-DNA interactome and chromatin structure association at different levels. First, we looked at whether our results were consistent within the same cell line (mESC) across different protocols (RADICL-seq and GRID-seq) or not. Figure 4A (left) shows the intersection of RNA sets with paired contacts in each protocol, with most of the results overlapping. When comparing only non-coding RNAs (right), the intersection is also significant, but smaller, likely due to the smaller number of contacts of non-coding RNAs, which complicates the comparative analysis. Figure 4B shows similar graphs comparing different mouse cell lines (mESC and mOPC) within the same protocol—RADICL-seq. The intersection is somewhat less significant than that between the results on different protocols within the same cell line, which may indicate the presence of tissue specificity in the RNA-DNA interactome association and the chromatin structure. Next, we analyzed more closely the conservativeness of paired contacts between experiments. We proposed a procedure (described in detail in the Materials and Methods section) using Fisher’s exact test. One experiment in the pair was selected as the reference, and the second as the target. The results of this for a pair of experiments, RADICL-seq and GRID-seq for mESC cells, are shown in Figure 4C. For most of the analyzed RNAs, there is a tendency toward greater conservation of paired contacts. Since this analysis was within the same cell line, the differences are mostly explained by noise and technical biases in the experimental protocols. Thus, it can be concluded that structurally associated pairs of contacts are less noisy than those lacking spatial proximity between the same RNA’s contacts. Next, we performed a similar analysis to compare different mouse cell lines (mESC and mOPC) of the RADICL-seq protocol (Figure 4D), and the results were quite similar.

Figure S9 shows paired contacts for RNA in the mESC protocols RADICL-seq and GRID-seq with significant conservation of paired contacts across lines for RNA Malat1 and ucaRNA X_17_3984_a_mm10. Despite the statistical significance in both cases (Fisher’s exact test p-value adjusted: 0), similar patterns of paired contact distribution on the chromosome were much more distinguishable for the ucaRNA, with many more peaks annotated for it than for Malat1 (24,441 vs. 396). These two examples indicate that RNA–chromatin interaction data are sparse, and, for highly contacting RNAs with many identified peaks, conservation of paired contacts is more likely to be observed. In contrast, for low-contact RNAs (Malat1 has many contacts but relatively few peaks due to its minor role in mESC [33]), this effect is underrepresented due to significant data incompleteness. Thus, this is another factor that causes underestimation of conservative cases.

The results of a similar analysis for human cell lines (K562 and hESC) are shown in Figures S10–S12.

### 2.4. RNA Contacts Are Associated with Chromatin Loops

Next, we analyzed how the linear distribution of RD-contacts is associated with chromatin loops. The CTCF protein, known to play a key role in the formation of chromatin loops, also contains an RNA-binding domain [34]. We investigated whether RNA–chromatin interactions from BaRDIC peaks are associated with chromatin loops. For the analysis, we used a background model constructed by shifting the coordinates of loop anchors while preserving their localization in the A/B compartments. Figure S13 shows the average number of RNA-DNA contacts around chromatin loop anchors (K562 Red-C). On the left, random regions are shown without compartment preservation, and, on the right, the A/B compartment ratio is preserved. In both cases, we observed overrepresentation of contacts at the loop anchors. However, when the distribution of loops across the A/B compartments was not considered, the random model underestimated the background number of contacts. This confirms that it is crucial to account for chromatin openness in such analyses, for instance, by preserving the number of observations in each A/B compartment.

**Figure 5:**
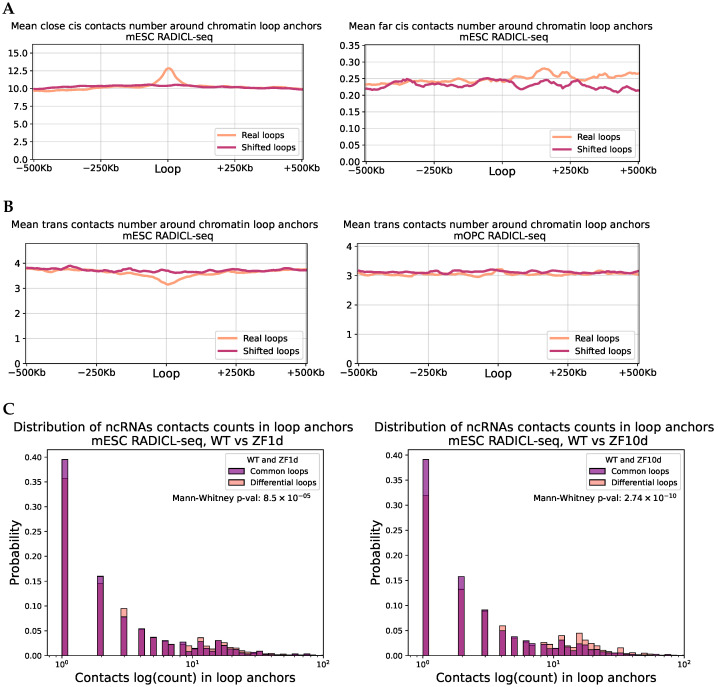
(**A**) The average number of RNA-DNA interactions in the vicinity of chromatin loop anchors: on the **left**—contacts closer than 20 Mb from the RNA gene; on the **right**—further, mESC RADICL-seq. (**B**) The average number of RNA-DNA trans-interactions in the vicinity of chromatin loop anchors: on the **left**—mESC RADICL-seq; on the **right**—mOPC RADICL-seq. (**C**) Comparison of the distributions of the number of RD-contacts of the ncRNA mESC RADICL-seq in the anchors of chromatin loops. Common loops—loops anchors present in both the WT and the mutant line. Differential loops—the anchors missing in the mutant cell line (on the **left** is a deletion in the ZF1 domain, on the **right** in the ZF10 domain).

We analyzed RNA contacts on gene chromosomes, dividing them into “close” (less than 20 Mb from the gene) and “distant” categories, as well as contacts on other chromosomes. RNA preferentially contacts chromatin loops primarily near its gene. RNA is generally indifferent to distant loops (Figure 5A, mESC RADICL-seq). This is likely due to RD-scaling, as more contacts are observed near the RNA gene. Overrepresentation of contacts at loop anchors near the RNA gene is observed for most protocols we considered; however, the mOPC cell line is an exception (Figure S14). In this case, distant contacts are also enriched at the loop anchors. Thus, we observed the tissue specificity in the interplay between RNA-DNA interactome and chromatin loops, which may indicate the functionality of this observation.

We separately considered only the trans-contacts, and Figure 5B shows the results for the RADICL-seq protocol as an example (mESC on the left, mOPC on the right). The result differs from long-range cis-interactions; in mOPC (Figure S14), there were no differences from the background, whereas in mESC, there was some underrepresentation of RNA contacts around the loop anchors. The results for mESC GRID-seq were consistent (Figure S14). We also conducted this analysis using the whole set of RNA-DNA interactions without BaRDIC peaks being taken into account. Such a prominent enrichment of contacts in loop anchors was not observed in most cases (Figure S15).

We aimed to identify which RNAs contribute to this result by using Fisher’s exact test. Using RADICL-seq data for mESCs, we found significant overrepresentation of contacts in chromatin loops for 2432 RNAs. Among the known long non-coding RNAs, there is Kcnq1ot1 (adjusted *p*-value: $1.6\times 10^{-11}$, odds ratio: 1.5).

As demonstrated in [34], deletions in the CTCF RNA-binding domain result in the disruption of a significant number of chromatin loops. The authors introduced deletions in two domains: ZF1 and ZF10. In the case of ZF1 deletion, most loops were disrupted, whereas fewer loops were affected by ZF10 deletion. We hypothesized that RNAs might show a greater tendency to interact with loops where the presence of the CTCF RNA-binding domain is critical (details in Supplementary Note S3, Figure S16). Figure S17 shows the distribution of the number of contacts at the loop anchors. We observed a small but significant inverse effect; RNA-DNA interactions were more abundant in loops that do not depend on the CTCF RNA-binding domain. However, when considering only the contacts of non-coding RNAs, the expected significant effect was observed (Figure 5C, with WT vs. ZF1d on the left and WT vs. ZF10d on the right). The data for mESC GRID-seq were consistent with the RADICL-seq results, except for the lack of significance for the ZF1 domain (Figures S17 and S18).

### 2.5. RNA-DNA Interactome and TADs Association

**Figure 6:**
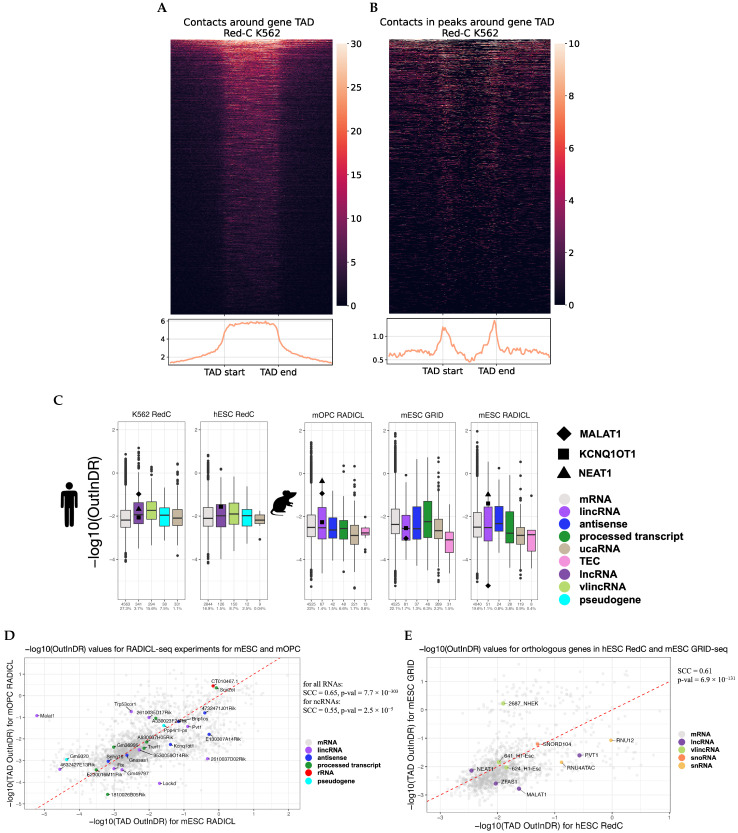
(**A**) Distribution of the full set of RD-contacts around the TADs of RNA genes (Red-C K562). (**B**) Distribution of RD-contacts from BaRDIC peaks around the TADs of RNA genes. (**C**) Value −log10(OutInDR), which reflects the tendency of RNA to extend beyond the boundaries of its TAD, for different RNA biotypes (in different colors) for different human and mouse cell lines. The numbers presented below the graphs indicate the quantity of RNA genes included in the analysis and the percentage of analyzed RNAs relative to RNAs found in the interactions data. (**D**) Scatterplot with values −log10(OutInDR) for the RADICL-seq experiments for the mESC cell line (on the X-axis) and the mOPC line (on the Y-axis). (**E**) Scatterplot with values −log10(OutInDR) of orthologous genes in the hESC (X-axis) and mESC GRID-seq (Y-axis) cell lines.

We analyzed how the distribution of RNA contacts with chromatin is affected by the TAD in which the gene of this RNA is located. In earlier studies, a similar analysis was carried out, but RD-scaling was not taken into account, the effect of which on RD-interactions is very significant. We selected the RNAs whose genes were located in the TADs and constructed the distribution of their contacts in the vicinity of the parental TADs. We did this both without taking RD-scaling into account (Figure 6A) and with RD-scaling considered (contacts from the peaks of the BaRDIC program, Figure 6B). We saw a radical difference in the observed patterns. This result is consistent with earlier work that did not take RD-scaling into account; the intensity of interactions dropped sharply at the TAD boundary, with the exception of the Red-ChIP on CTCF (see the next section, Figure S19), where small peaks were observed at the TAD boundaries. When RD-scaling was taken into account, contacts were still overrepresented as a whole inside the TAD (shown below); however, significant peaks were observed at the boundaries of the TADs. Thus, RNA tends to interact with the boundaries of the TADs. This effect is explained by more active transcription at the boundaries of than inside TADs [16,35], and RNA contacts are more associated with active chromatin.

We investigated the propensity of different RNA biotypes to extend beyond the boundaries of their TADs (Figure 6C). Following the application of filtering procedures and the identification of significant interactions, it was observed that, for some biotypes, only a small number of RNAs were included in the analysis (e.g., antisense RNA and TEC). Consequently, the limited data quality precludes definitive conclusions regarding the global behavior of these RNA biotypes.

To characterize the tendency of RNA to be distributed along the chromosome, we used the ratio of peaks contact densities outside versus inside the TAD (hereinafter referred to as OutInDR). This value increases with the RNA’s tendency to leave the parental TAD and does not depend on the level of gene expression. We found that some RNA biotypes, such as lncRNAs and vlincRNAs, were more prone to interact outside parental TADs compared with mRNAs in K562 cells (Figure 6C). The p-values from the two-sided Mann–Whitney test varied from $9.9\times 10^{-7}$ to $2.2\times 10^{-21}$ in our experiments, as presented in Table S4. In the K562 and mOPC cell lines, we observed the expected pattern; KCNQ1OT1 RNA mainly functions at the locus of its gene [29], while MALAT1 and NEAT1 do not. We also observe that, while MALAT1 RNA does not have a pronounced functional role in mESC [33], it is mainly localized at the site of synthesis on its chromosome. However, when MALAT1 RNA participates in the differentiation of mOPC [36], it starts to extend beyond its TAD and contact the boundaries of other TADs (*p*-values of the Fisher’s exact test are $1.5\times 10^{-66}$ and $6\times 10^{-30}$, Figure S20). During this differentiation, NEAT1 RNA begins to cross the boundaries of its TAD and interact with other TADs (the p-value of the Fisher’s exact test is $7.7\times 10^{-26}$, Figure S20).

To study the differences in the propensity of RNAs to contact regions outside their parental TAD in different protocols and cell lines, we analyzed the GRID-seq experiments for mESC and RADICL-seq for mESC and mOPC. We obtained a set of 1920 genes, with a sufficient number of significant contacts found in all three experiments. The correlation of the OutInDR value (full set of RNAs) between different protocols within the same cell line was higher than between different cell lines for the same protocol, indicating a tissue-specific tendency of RNAs to interact with regions outside their parental TADs (Figure 6D, Figure S21). ncRNAs exhibit more conservative behavior within the same organism and more pronounced specificity in different cell lines compared to mRNAs (for mRNA GRID-seq mESC and RADICL-seq mOPC, SCC = 0.88, *p*-value = $2.7\times 10^{-17}$; for ncRNA RADICL-seq mESC and RADICL-seq mOPC, SCC = 0.55, *p*-value = $2.5\times 10^{-5}$).

To study the conservativeness of OutInDR values across humans and mice, we compared the Red-C protocol data for hESC with the GRID-seq and RADICL-seq data for mESC. A list of 1283 orthologous genes was obtained. Then, we compared the OutInDR value for each gene in the hESC and mESC cell lines (Figure 6E, Figure S22). We observed conservativeness in the tendency of orthologous human and mouse RNA to contact areas outside their parental TADs (for GRID-seq: SCC = 0.61, *p*-value = $6.9\times 10^{-131}$; for RADICL-seq: SCC = 0.59, *p*-value = $6.2\times 10^{-120}$).

We compared the OutInDR values of the lincRNA Malat1 between the “all-to-all” and “one-to-all” experimental data. A comparison of the “all-to-all” data of the GRID-seq experiment for mESC and the data of seven “one-to-all” experiments (Figure S23) showed that Malat1 exhibited a greater tendency to leave its TAD in the “one-to-all” experiments compared to the “all-to-all” data (the value of log10(OutInDR) in the experiment GRID-seq for mESC was −2.77, the median log10(OutInDR) value in the “one-to-all” experiments was −1.07). In an experiment where cells were treated with a transcription elongation inhibitor [37], there was a significant decrease in Malat1’s ability to interact with areas outside its TAD, which was expected due to Malat1’s role in speckles.

### 2.6. Analysis of RNA–DNA–Protein Interactions

An experimental protocol, Red-ChIP, has been proposed which allows researchers to fix RNA-DNA interactions with an additional step of immunoprecipitation for a specific protein [38]. In this study, four datasets were obtained: one from K562 cells with immunoprecipitation for CTCF protein, one from hESC cells with immunoprecipitation for EZH2 (a component of the polycomb repressive complex), and two additional Red-C datasets (as input) for the same cell lines. We repeated the above analysis to determine the association of RNA with chromatin structure and identified many significant RNAs, both for cis- and trans-RD-interactions (Figure S2).

**Figure 7:**
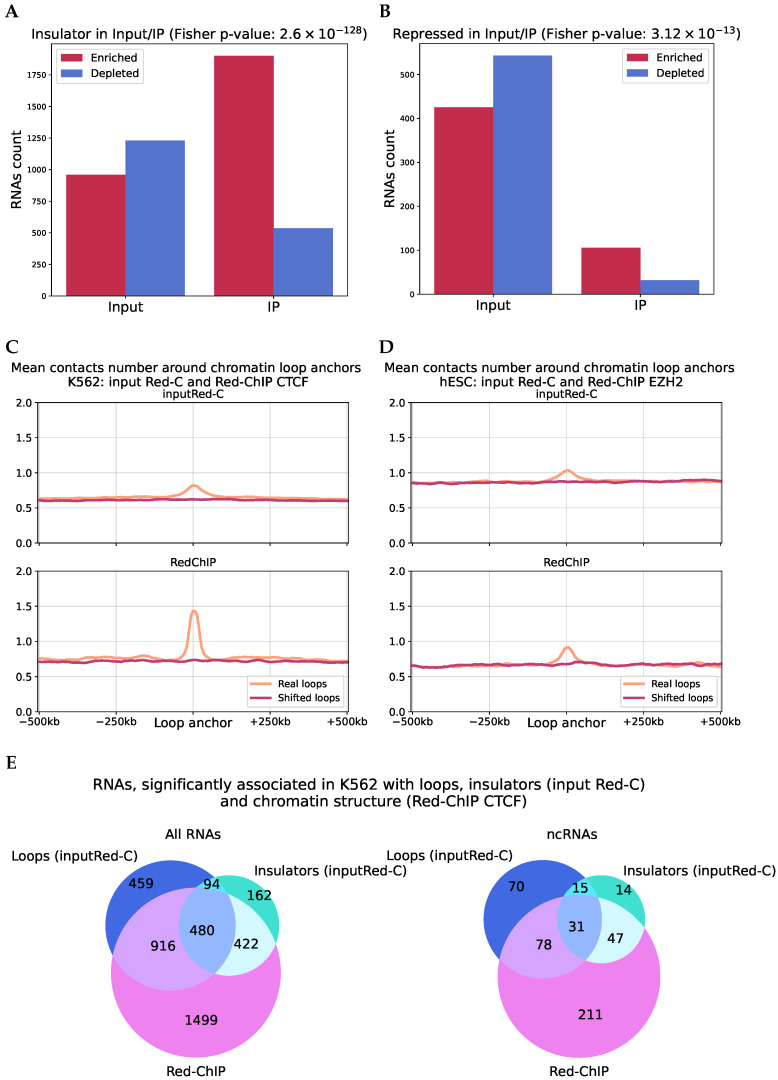
(**A**,**B**) The number of cases of significant enrichment and depletion of paired contacts in pairs of states in Red-C and Red-ChIP. (**C**) Comparison of the representation of contacts within the chromatin loop anchors (K562): **bottom row**—Red-ChIP on CTCF; **top row**—Red-C (the number of contacts is normalized to the number of significant contacts in the experiment). (**D**) Comparison of the representation of contacts within the chromatin loop anchors (hESC): **bottom row**—Red-ChIP on EZH2; **top row**—Red-C (the number of contacts is normalized to the number of contacts from peaks in the experiment). (**E**) Comparison of RNAs sets associated with chromatin structure, chromatin loops, and insulators. Numbers of intersection of RNAs significantly associated with loops (input Red-C data, K562) and with the chromatin structure (Red-ChIP on CTCF), and RNAs whose paired contacts are associated with insulators (input-Red-C) are shown. **Left**—all RNAs, **right**—ncRNAs.

We annotated paired contacts with pairs of ChromHMM states. The insulator state is similar to CTCF peaks, while the repressed chromatin state is characterized by peaks of PRC2 complex proteins. The number of RNAs whose paired contacts are enriched or depleted in the insulator states in both the input and Red-ChIP datasets (on CTCF) is shown in Figure 7A (similar for RedChIP on EZH2 in the repressed state in Figure 7B). We observed significantly greater representation of paired contacts in the corresponding chromatin states for Red-ChIP. At the same time, for example, paired contacts in RedChIP on EZH2 are depleted in the state of active transcription (Figure S24).

We also studied the protocol differences in the context of chromatin loops. For contacts fixed via CTCF, we observed a much clearer and higher peak in the loop anchors compared to conventional Red-C (Figure 7C), which is completely consistent with the role of CTCF in the organization of chromatin loops. For immunoprecipitation via EZH2, no such prominent peak was observed (Figure 7D) since this protein is not involved in the formation of chromatin loops. Additionally, for Red-ChIP on CTCF, we observed an association with chromatin loops not only for contacts near the gene, but also for distant contacts and trans-contacts, in contrast to the data from Red-C (Figure S14).

To isolate RNAs consistently associated with loops and CTCF, we compared the results of the analysis of paired contact enrichment with insulator states (input Red-C), RNA association with loop anchors (input Red-C), and RNAs associated with the chromatin structure in the Red-ChIP protocol (CTCF, K562 cell line). We found a significant number of intersections (Figure 7E). We selected those RNAs whose results intersect on specific chromosomes and that also overlap with RNAs identified from another Red-C experiment (Figure S25): LINC01184 (DNA parts: chr5), GARS1-DT (chr7), AC073529.1 (chrX), 1840_HUVEC (chr7), 1366_HepG2 (chr7)). These RNAs are the most likely to be involved in the regulation of CTCF.

## 3. Discussion

For a significant number of non-coding RNAs, their role in chromatin regulatory processes, structural organization, and interactions with specific proteins is well established. The number of datasets of RNA-DNA interactions is constantly growing, expanding the possibilities for analyzing the functions of chromatin-associated RNAs. In the HiChIRP [20] protocol, the first joint experimental study of RNA–chromatin interactions and the spatial structure of chromosomes for several RNAs in the “one-to-all” mode was performed. In this work, we applied a similar in silico analysis for genome-wide “all-to-all” data. We identified RNAs associated with chromatin structure; moreover, we showed that this property appears to be universal for many RNAs, which allows us to conclude that RNAs are localized along the genome in a manner that aligns with the chromosomal structure, rather than linearly. Furthermore, RNA contacts are not independent events; their distribution depends on the pairwise proximity of the corresponding genome loci. We observed this effect both within the same chromosome and between different chromosomes. We analyzed this effect on eight sets of experimental data from different protocols, cell lines, and organisms, showing consistent results. Moreover, we showed that there is a conservation between different experiments. Within a single cell line, most RNAs tend to interact with the same chromosomal structures, which indicates that these pairs of interactions are less noisy. Conservation is also observed between different cell lines. It is also important to note that the RNA–chromatin interaction data were significantly incomplete, making comparisons across different datasets difficult.

We analyzed the chromosomal structures with which individual RNAs are associated in the context of functional chromatin annotations. We found that the preferences of RNAs for specific chromosomal structures differ. For RNAs with known functions, we observed consistent results; MALAT1 preferentially interacts within speckles and active chromatin, while KCNQ1OT1 is enriched in repressed chromatin structures. Small nuclear RNAs are enriched in active chromatin states.

All of this highlights the importance of considering chromatin structure in the analysis of RNA–chromatin interactions. Although early studies focused on the influence of chromatin openness and the effect of RD-scaling on these data, the role of spatial factors in the distribution of RNA–chromatin interactions has not been comprehensively studied until now. We demonstrated its significant role in the different examples.

We also analyzed how chromatin loops are related to RNA–chromatin interactions. Our analysis shows that, in general, RNAs tend to contact the anchors of the loops closest to the RNA gene, and this pattern was observed for all datasets. It was further confirmed by the fact that, in the RedChIP experiment data, which included an immunoprecipitation stage with CTCF, this effect was especially obvious. Additionally, we analyzed Hi-C data for a mESC cell line with a mutated CTCF RNA-binding domain. We assumed that, since many chromatin loops disappear, we may see differences in the distributions of RD-contacts with these loops. This hypothesis was not confirmed for a full RNA set, but such an effect was shown for ncRNAs.

We also conducted a comprehensive analysis of the relationship between RNA–chromatin interactions and TADs. First, we showed that it was critically important for this analysis to take into account RD-scaling, since this showed the preference of RNA for interacting with the boundaries of TADs. We showed that individual RNA biotypes exhibit different localization patterns on chromatin relative to their TAD, and found that these patterns are mostly preserved across different protocols, cell lines, and even organisms (humans and mice). However, we also observed some degree of cellular specificity in this behavior, suggesting that, although the tendency of RNA to localize beyond its TAD is conserved, it may also be influenced by tissue-specific factors. We found a change in the localization pattern of MALAT1 RNA as it differentiated from mouse embryonic stem cells into oligodendrocyte progenitor cells. In mESC, it is localized at the site of synthesis, but, as it acquires a functional role in mOPC, it begins to extend beyond its parental TAD and contact the boundaries of other TADs located in more distant parts of the genome. Analysis of orthologous genes in human and mouse embryonic stem cells revealed a significant positive correlation, indicating that the tendency of RNAs to bind to regions outside their parental TADs is partially preserved between species.

We also found previously uncharacterized non-coding RNAs that may be associated with chromatin structure.

## 4. Materials and Methods

### 4.1. Procedure for Identifying Associations Between RNA-DNA and DNA-DNA Interactions

The contact tracks of each individual RNA were binned according to the Hi-C map bin size (10 Kb for intrachromosomal interactions and 50 Kb for interchromosomal interactions). A pair of RNA–chromatin contacts was considered structurally associated if there was a significant interaction between the corresponding bins of the Hi-C map (based on FitHiC2, Figure 1). To assess whether there was an overrepresentation of such pairs, we used a random model: a Hi-C map shifted along each axis (along the main diagonal for the intrachromosomal case) by a given number of nucleotides, taking into account the A/B compartments (Figure 1, details in Supplementary Note S4; Figures S1 and S26). In this study, a 2 Mb shift was chosen. Next, the number of such pairs was calculated for each chromosome (for each pair of chromosomes in the case of interchromosomal interactions) and for each RNA independently. The obtained *p*-values from Fisher’s exact test were adjusted for multiple testing using the Benjamini–Hochberg correction (separately for the intrachromosomal and interchromosomal cases). We considered RNAs with a q-value less than 0.05 to be significantly associated with chromatin structures on a given chromosome (or on a given pair of chromosomes in the case of the interchromosomal interaction analysis). The code used for the analysis can be found at https://github.com/ZvezdinDmitry/RNA_DNA_HiC (access date: 30 November 2024).

### 4.2. Data Sources

The human gene annotation was compiled from GENCODE (release 35, grch38.p13) [39], small and transport from UCSC [40], and vlinc RNA from the original work on their annotation [41]. For the mice, GENCODE mm10.p6 was used and small and transport from UCSC. The annotation of the hypothetical ucaRNAs was taken from the RNA-ChromDB [42] database. Hi-C maps with pre-calculated normalization weights in cool format, as well as the annotation of the A/B compartments, were taken from the 4DNucleome database (K562: 4DNESI7DEJTM, hESC: 4DNES2M5JIGV, mESC: 4DNESDXUWBD9, mOPC: 4DNESJ9SIAV5) [43]. Hi-C data for cell lines with deletions in CTCF RNA-binding domains were taken from the study of the effect of the CTCF RNA-binding domain on chromatin structure [34]. The chromatin states ChromHMM annotation was taken from the original work on ChromHMM [44], and the SPIN states annotation was taken from the work on SPIN states [26].

### 4.3. Data Preprocessing

All data manipulations were performed using Python 3.10.13. For intersections and other manipulations with genomic intervals, bedtools (v2.31.0) [45] and bioframe (v0.5.0) [46] were used. Only those RNA-DNA contacts that fell into the peaks of the corresponding RNAs were taken for analysis unless otherwise stated. The peaks were obtained using the program BaRDIC with the default parameters for “all-to-all” protocols. BaRDIC performs peak calling with RD-scaling correction for cis-interactions. The scaling factor is calculated separately for each RNA and depends on the distance between the source gene of the RNA and the current bin. Its profile is smoothed using the cubic spline [21]. The threshold for peak selection is set so that a given proportion of RNA–chromatin contacts (10%) falls into peaks (details in Supplementary Note S5) [47]. For the analysis of TADs, only intrachromosomal RNA-chromatin contacts were used, so the threshold was selected separately using only them. For “one-to-all” protocols, we used the parameters -trans_min 1000 -cis_start 100 –trans_step 100. The threshold was selected similarly to the “all-to-all” data. Contacts within the BaRDIC peaks were used for the analysis. For basic manipulations with Hi-C data, the libraries cooler (v0.9.3) [48] and cooltools (v0.5.4) [49] were used. Significant interactions in the Hi-C data were obtained using the FitHiC2 (v2.0.8) [50] program for intrachromosomal interactions with a bin size of 10 Kb (run separately for each chromosome, FDR: 0.05), and for interchromosomal interactions—50 Kb (FDR: 0.2). Normalization weights were calculated for the maps using the KR algorithm, using the script included in the FitHiC2 package.

### 4.4. Analysis of Functional Chromatin Annotations

The ChromHMM annotation for K562 and hESC cells was converted from the hg18 version of the human genome to the hg38 genome assembly using the liftover [51] web service. ChromHMM and SPIN states were grouped according to tables (Tables S5 and S6) to increase their genome coverage and reduce noise level. We considered the SPIN state of Interior_Repr2 separately as a NAD_like, since the authors indicated that these regions tend to aggregate with the nucleolus [26].

To synchronize chromatin annotations and bin pairs of interactions, we used an approach inspired by the work on Multiplex GAM [52]. We assumed that a given bin belongs to a given state if at least one base pair falls within it; therefore, a bin can belong to several different states within the same annotation. A pair of bins is assigned to a given state if both bins are in that state. For each RNA and each chromosome, we independently counted the number of paired contacts between each pair of chromatin states with the same label. To account for the varying proportions of significant Hi-C interactions between pairs of loci, we calculated the expected number of paired contacts for a given chromatin state based on the proportion of Hi-C peaks between loci within that state. The significance of the differences between the observed and expected fractions was assessed using the chi-square test.

### 4.5. Comparative Analysis of Protocols

To assess how the spatial association of a given RNA is conservative between different experiments, we used Fisher’s exact test with a two-sided alternative hypothesis. We designated one experiment as a target experiment, and the other as the reference experiment. The first category in the contingency table corresponds to whether the contacts in the target experiment are paired or not, based on the Hi-C map. The second category is based on the reference experiment, where the question is whether the pair was present in the reference experiment. Next, contact pairs in these categories were counted for each RNA in the target experiment and it was determined whether there were differences in the preservation of structurally associated and other contact pairs between experiments. Then, the target and reference experiments were reversed. For further analysis, only cases of significance in both cases and deviations in one direction were taken (odds ratio statistics).

### 4.6. Chromatin Loops

Chromatin loops were annotated using the dots function of the cooltools [49] package with default parameters (which implements an algorithm similar to HiCCUPS [17]) at a 10 Kb bin size. The expected values were also calculated using cooltools. Next, the annotation of the pairs of coordinates of the loops was reduced to the linear coordinates of the loop anchors. To determine the RNAs associated with loop anchors with Fisher’s exact test, the loop anchors were expanded to 5 bins (50 Kb). We used a background model by shifting the loop anchors by 2 Mb while maintaining the regions belonging to A/B compartments to account for chromatin openness. The test was performed for each RNA separately for cis- and trans-RD-contacts, after which an adjustment for multiple testing was made using the Benjamini–Hochberg procedure (threshold for q-value = 0.05). For loop-level analysis and plotting, the neighborhood around the loop anchors was set to 500 Kb upstream and downstream.

Datasets of Hi-C mESC cells with CTCF mutated RNA-binding domains were converted to cool format and preprocessed (normalized, calculated expected, annotated loops) by cooler and cooltools libraries. Loops were classified as dependent on the CTCF RNA-binding domain if no loops were found in the dataset without the CTCF RNA-binding domain within 20 Kb. Otherwise, the loops were considered independent of the CTCF RNA-binding domain.

### 4.7. TADs

TADs were annotated using the TopDom (v0.10.1) [53] tool, selected based on a comparative analysis [54]. The annotation was carried out on a normalized Hi-C map with a 10 Kb bin size, and the window size was set to 20. We determined RNAs’ parental TADs by intersecting the coordinates of the RNA genes with the coordinates of the TADs (if the gene intersected with the TAD that was more than half its length). To demonstrate the effect of RD-scaling around the TAD in which the RNA gene was located, a neighborhood equal to the length of the TAD was selected, and all TADs were reduced to a single length. The top 100 RNAs were selected according to the number of contacts in the selected region on each chromosome.

According to the position of the DNA part of the contact relative to the TAD of the gene, the contact was classified as either intra-TAD or extra-TAD. To assess the propensity of the RNA to interact outside its TAD, the OutInDR parameter was used, defined as the ratio of the density of contacts outside the TAD to the density of contacts inside the TAD (InOutDR—inner/outer densities ratio). Only genes with at least 10 cis-chromosomal contacts were considered.

To search for orthologs, we used the program ortho2align (v1.0.5) [55]. The found orthologs were filtered by a Jaccard index of more than 0.5. To compare the propensity of human and mouse orthologous RNAs to leave their TAD, softer thresholds were applied during the preprocessing stage, specifically, 20% of contacts should be within peaks. Experiments for different organisms were compared (input RedC for hESC and GRID-seq and RADICL-seq for mESC).

For a set of “one-to-all” experiments, peaks of lincRNA Malat1 interactions were obtained using BaRDIC. Next, the OutInDR value was calculated.

## Data Availability

Hi-C data were taken from the 4DNucleome database (K562: 4DNESI7DEJTM, hESC: 4DNES2M5JIGV, mESC: 4DNESDXUWBD9, mOPC: 4DNESJ9SIAV5) and GEO: GSE125595. RNA-DNA interactomes were taken from RNAChromDB (ID for “one-to-all” experiments: 36, 49, 52, 55, 58, 92, 93, 94, 156, 198).

## Supplementary Materials

Supporting information can be downloaded at https://www.mdpi.com/article/10.3390/ijms26031137/s1.

## Abbreviations

The following abbreviations are used in this manuscript:

RD	RNA-DNA
lncRNA	Long non-coding RNA (human)
vlincRNA	Very long intergenic non-coding RNA (human)
lincRNA	Long intergenic non-coding RNA (mouse)
ucaRNA	Unannotated chromatin-associated RNA
snRNA	Small nuclear RNA
snoRNA	Small nucleolar RNA
TAD	Topologically associated domain
mESC	Mouse embryonic stem cells
mOPC	Mouse oligodendrocytes progenitor cells
hESC	Human embryonic stem cells
OutInDR	Contacts densities ratio outside versus inside the TAD
