# Supplementary material for: A Joint Analysis of RNA-DNA and DNA-DNA Interactomes Reveals Their Strong Association
Source: Int J Mol Sci. 2025 Jan 28;26(3):1137. doi: 10.3390/ijms26031137 (PMC11817408; doi:10.3390/ijms26031137)
Supplement: Supplementary file 1 [file ijms-26-01137-s001.zip › rnadna_hic_paper_supplementary_information.pdf]

Supplementary Materials: Joint analysis of RNA-DNA and DNA-DNA interactomes reveals their strong association

List of Figures

S1. Paired Rnu3a RNA contacts (mESC RADICL-seq) based on real (left) and background (right) Hi-C maps. The number of paired contacts in the background model is significantly lower (p-value adjusted: 2.3×10^{-95}, odds ratio: 4.5), which clearly confirms the validity of our procedure. . . . 4
S2. Distribution of significant results by RNA types (continued on the next page). . . . 5
S2. Distribution of significant results by RNA types 6
S3. Examples of paired contacts of lncRNA of mOPC RADICL-seq data: Malat1 (chr15) on the left, Pvt1 (chr15) on the right. . . . 7
S4. Examples of paired contacts of lncRNA of mOPC RADICL-seq data: Neat 1 (chr2) on the left, Kcnq1ot1 (chr7) on the right. . . . 7
S5. Examples of interchromosomal paired contacts of long non-coding RNAs (mESC RADICL-seq). . . . 8
S6. An example of the effect of chromosomal rearrangements of the K562 cell line on interactomes: Hi-C data on the left, Red-C data on the right. Brighter color corresponds to higher interactions frequency. . . . 9
S7. The enrichment of paired contacts of MALAT1 RNA in chromatin annotations on different chromosomes. . 10
S8. tSNE is colored by enrichment of paired contacts of non-coding RNAs in various chromatin state (continued on the next page). . . . 11
S8. tSNE is colored by enrichment of paired contacts of non-coding RNAs in various chromatin state (continued on the next page). . . . 12
S8. tSNE is colored by enrichment of paired contacts of non-coding RNAs in various chromatin state 13
S9. Examples of paired contacts of RNA from the protocols RADICL-seq (left) and GRID-set (right) of the mESC cell line. The top row is Malat1. The bottom row is X_17_3984_a_mm10. . . . 14
S10. The intersection of sets of RNAs associated with the chromatin structure between the Red-C and Red-C (input) protocols, cell line K562, on the left - all RNAs, on the right - non-coding RNAs. . . . 15
S11. The intersection of the sets of RNAs associated with the chromatin structure between the protocols of the K562 and hESC cell lines, the Red-C (input) protocol, on the left - all RNAs, on the right - non-coding RNAs. 15
S12. The relationship of the q-values and the odds ratios of the Fisher's exact test. On the left - comparison of Red-C and Red-C (input) experiments, on the right comparison of K562 and hESC cell lines. . . . 16
S13. The average number of RNA-DNA (K562 Red-C) interactions in the vicinity of chromatin loop anchors, on the left the background model is random shift, on the right the background model is shift, taking into account belonging to the A/B compartment. . . . 16
S14. The average number of RNA-DNA interactions in peaks in the vicinity of chromatin loop anchors (continued on the next page). . . . 17
S14. The average number of RNA-DNA interactions in peaks in the vicinity of chromatin loops anchors. . . . 18
S15. The average number of the whole set of RNA-DNA interactions in the vicinity of chromatin loop anchors (continued on the next page). . . . 19
S15. The average number of the whole set of RNA-DNA interactions in the vicinity of chromatin loops anchors. 20
S16. Examples of annotated loops on Hi-C maps in different conditions, on the left - WT, in the center - deletion in ZF10, on the right - deletion in ZF1 (chr17:30330000:32900000). Brighter color corresponds to higher interactions frequency. . . . 21
S17. Comparison of the distribution of the number of RD-contacts in mESC within the anchors of chromatin loops. Common loops are found in both WT and mutant lines. Differential loops are absent in the mutant cell line (on the left is a deletion in the ZF1 domain, on the right is in the ZF10 domain, the upper row is RADICL-seq, the lower one is GRID-seq). . . . 21
S18. Comparison of the distribution of the number of ncRNAs RD-contacts in mESC GRID-seq within the anchors of chromatin loops. Common loops are found in both WT and mutant lines. Differential loops are absent in the mutant cell line (on the left is a deletion in the ZF1 domain, on the right is in the ZF10 domain. 22
S19. Left - distribution of the full set of RD-contacts around the TADs of RNAs genes (Red-C K562), right - distribution of RD-contacts from BaRDIC peaks around the TADs of RNAs genes (continued on the next page). . . . 23
S19. Left - distribution of the full set of RD-contacts around the TADs of RNAs genes (Red-C K562), right - distribution of RD-contacts from BaRDIC peaks around the TADs of RNAs genes (continued on the next page). . . . 24

S19. Left - distribution of the full set of RD-contacts around the TADs of RNAs genes (Red-C K562), right - distribution of RD-contacts from BaRDIC peaks around the TADs of RNAs genes (continued on the next page). 25
S19. Left - distribution of the full set of RD-contacts around the TADs of RNAs genes (Red-C K562), right - distribution of RD-contacts from BaRDIC peaks around the TADs of RNAs genes. 26
S20. RD-contacts distribution in TADs and boundaries in different cell lines. Top row - MALAT1 contacts, bottom row - NEAT1. 28
S21. Scatterplot with values -log10(OutInDR) for the RADICL-seq experiment on the X axis and GRID-seq on the Y axis for the mESC cell line. 28
S22. Scatterplot with values -log10(OutInDR) of orthologous genes in the hESC (X-axis) and mESC RADICL-seq (Y-axis) cell lines. 29
S23. Comparison of the -log10(OutInDR) value for lincRNA Malat1 in "one-to-all" and "all-to-all" experiments for mESC GRID-seq. 29
S24. The number of cases of significant overrepresentation and underrepresentation of paired contacts in pairs of the active transcription state in Red-C and RedChIP EZH2 (hESC). 30
S25. The intersection of RNAs which are significantly associated with loops (Red-C data), with the chromatin structure (Red-ChIP), and whose paired contacts are associated with insulators (Red-C). 30
S26. The intersection of the results of significant association of RNA-DNA interactions and chromatin structure depending on the shift (K562 Red-C). The numbers of different results are close between the shift values, which indicates that the magnitude of the shift does not affect the degree of difference between the real model and the background one. 31

List of Tables

S1. Characteristics of RD-protocols. 4
S2. Non-coding RNAs (mESC RADICL-seq) associated with chromatin structure only at the interchromosomal level. RNAs associated with more than 4 pairs of chromosomes are shown. 8
S3. The number of chromosomes (pairs of chromosomes for the interchromosomal case) on which there is an association of contacts with the chromatin structure (statistics were calculated on peaks counts, due to their small width and high coverage of “one-to-all" data). 9
S4. A two-sided Mann-Whitney test for OutInDR values of different RNA biotypes and mRNA (NS - non significant, adj p-value > 0.05). 27
S5. ChromHMM states grouping [1]. 31
S6. SPIN states grouping [2]. 32

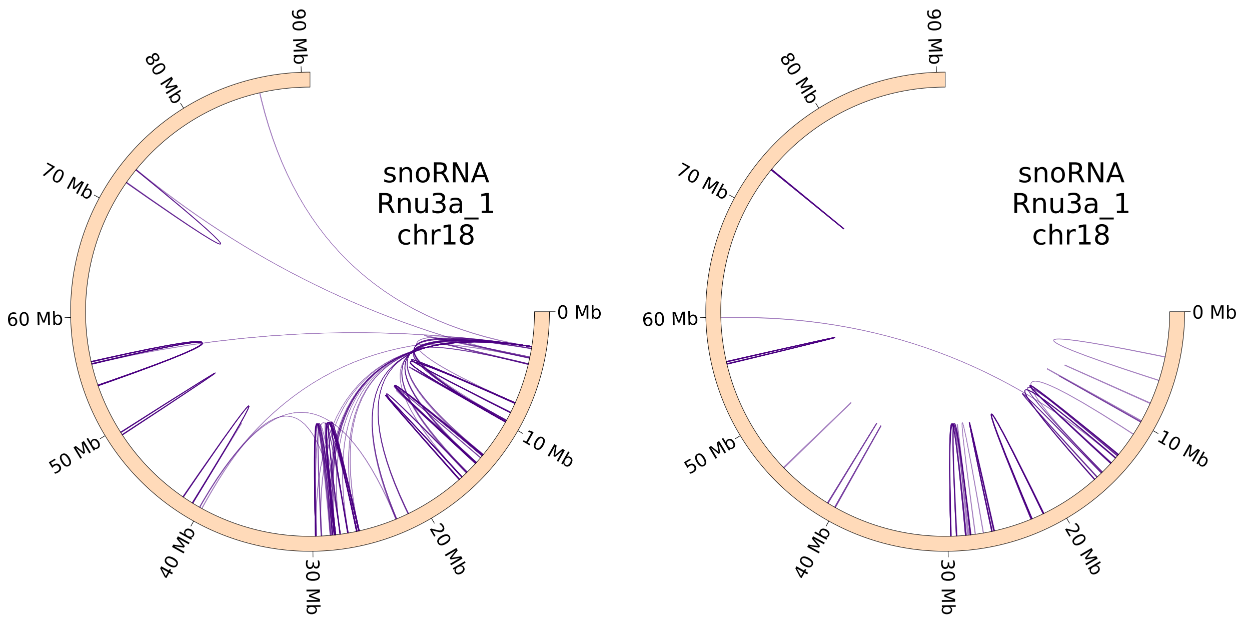

Figure S1. Paired Rnu3a RNA contacts (mESC RADICL-seq) based on real (left) and background (right) Hi-C maps. The number of paired contacts in the background model is significantly lower (p-value adjusted: 2.3×10^{-95}, odds ratio: 4.5), which clearly confirms the validity of our procedure.

Table S1. Characteristics of RD-protocols.

Protocol	Paper	Cell line	Interactions number	Interactions set	BaRDIC peaks count	FDR threshold
Red-C	[3]	K562	43603491	All<br>Cis	663396<br>91661	0.203<br>0.11
Red-C (input)	[4]	K562	29288865	All<br>Cis	942379<br>59040	0.015<br>0.003
Red-C (input)	[4]	hESC	16158007	All<br>Cis	238727<br>45194	0.119<br>0.055
Red-ChIP	[4]	K562	19323288	All<br>Cis	645219<br>40878	0.096<br>0.012
Red-ChIP	[4]	hESC	4041758	All<br>Cis	75271<br>21843	0.048<br>0.048
RADICL-seq	[5]	mESC	47319620	All<br>Cis	564435<br>54605	0.171<br>0.001
RADICL-seq	[5]	mOPC	45974349	All<br>Cis	522858<br>60127	0.187<br>0.007
GRID-seq	[6]	mESC	62398550	All<br>Cis	443485<br>73272	0.02<br>0.00015

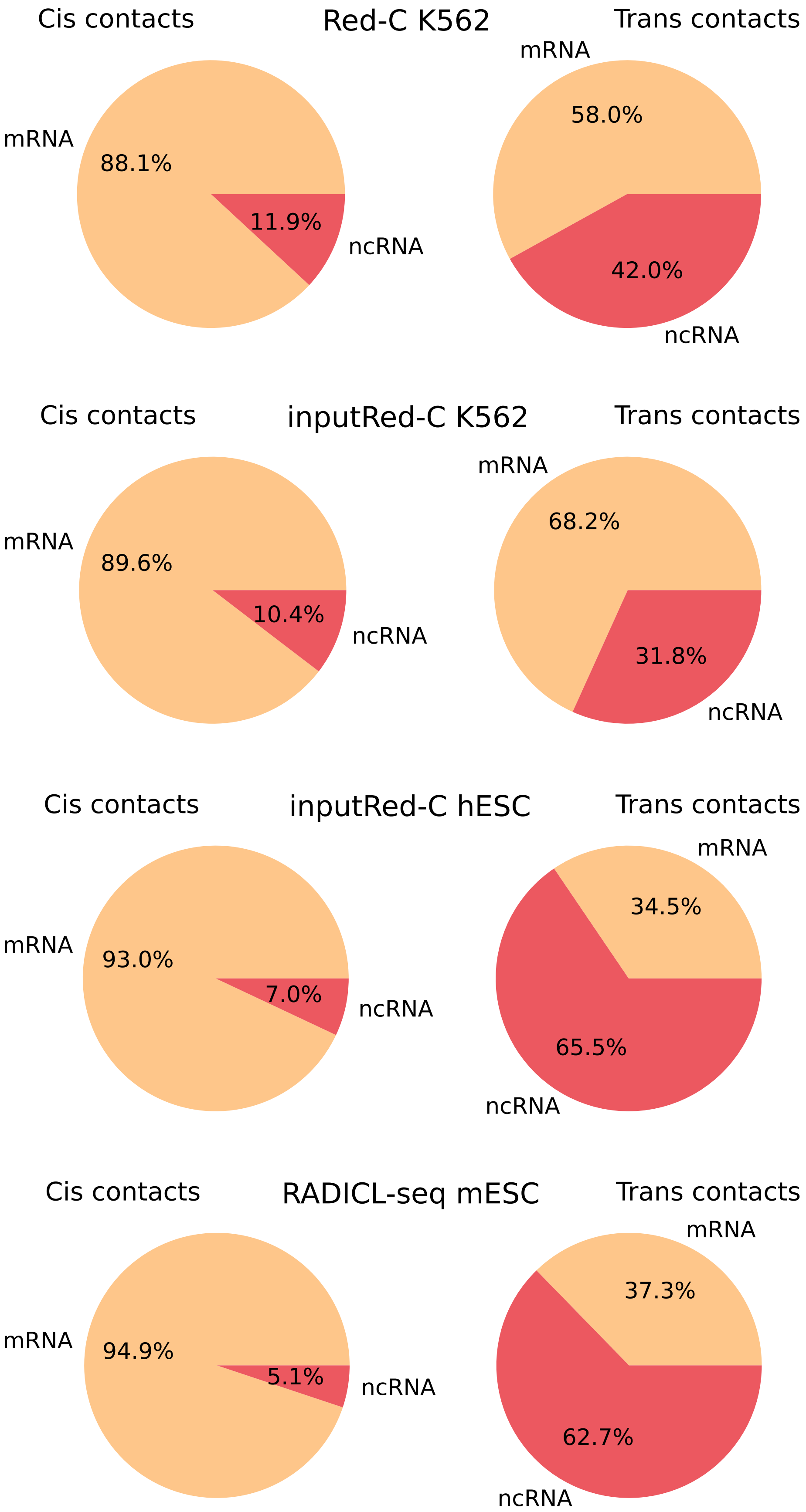

Figure S2. Distribution of significant results by RNA types (continued on the next page).

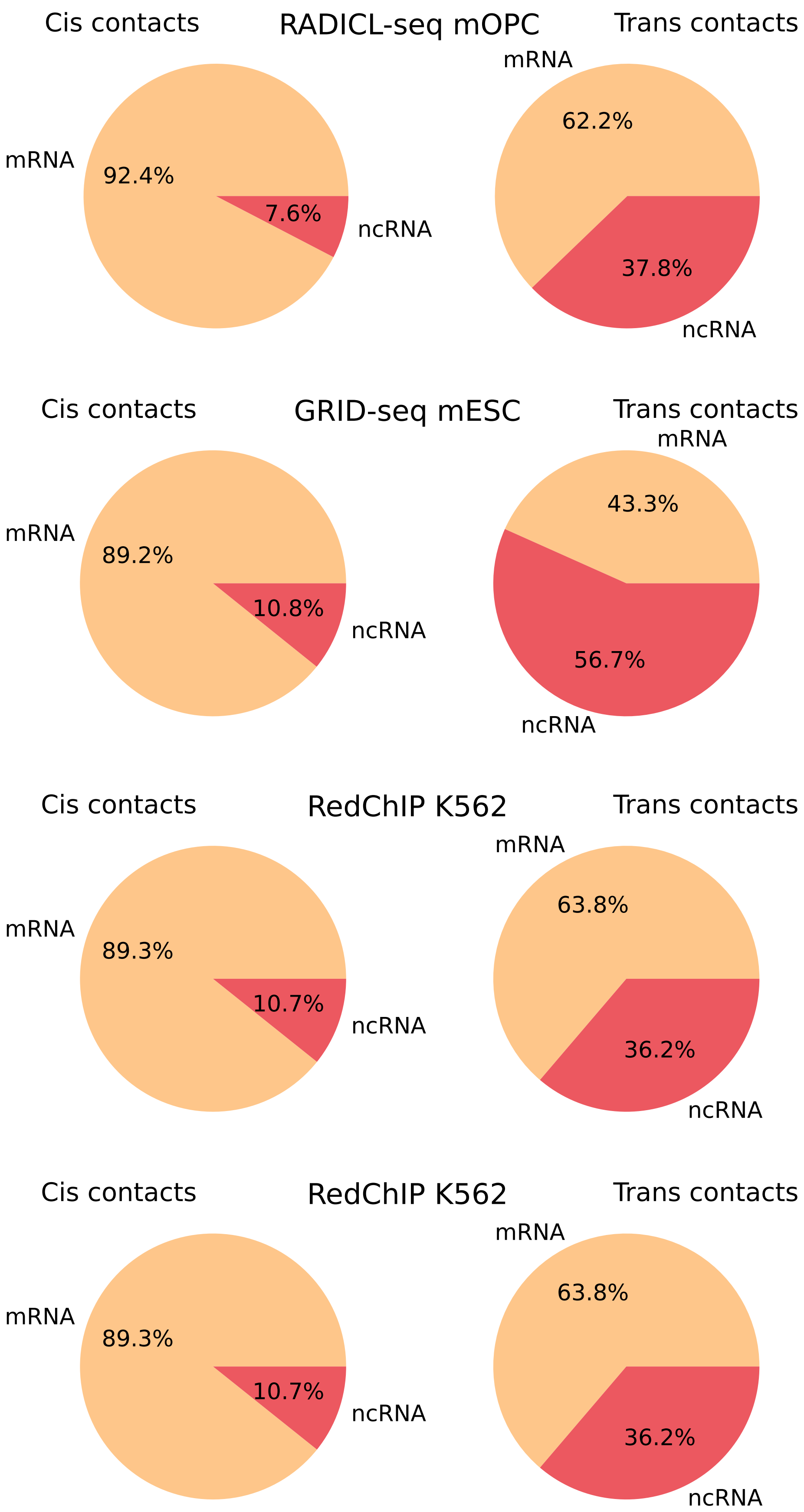

Figure S2. Distribution of significant results by RNA types

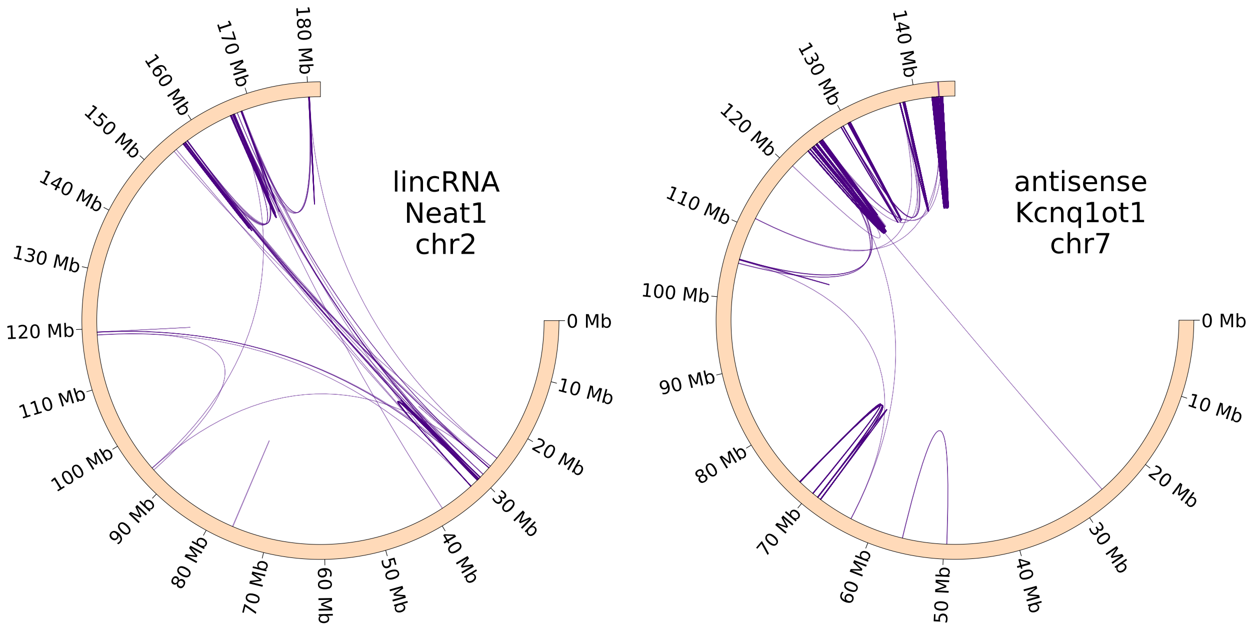

Figure S3. Examples of paired contacts of lncRNA of mOPC RADICL-seq data: Malat1 (chr15) on the left, Pvt1 (chr15) on the right.

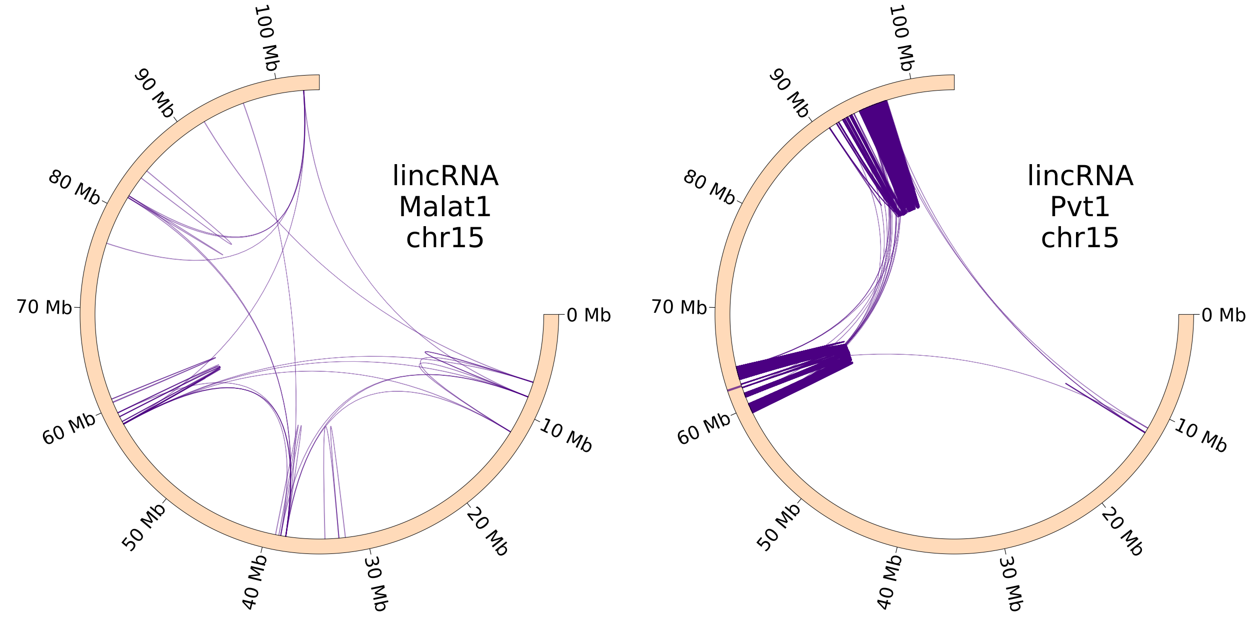

Figure S4. Examples of paired contacts of lncRNA of mOPC RADICL-seq data: Neat 1 (chr2) on the left, Kcnq1ot1 (chr7) on the right.

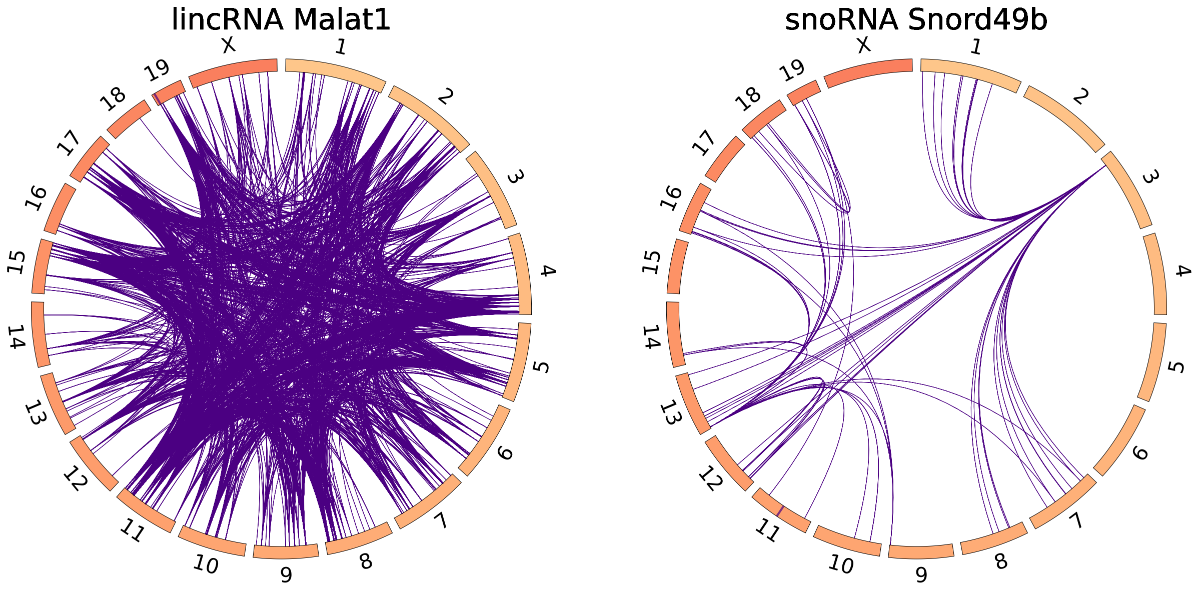

Figure S5. Examples of interchromosomal paired contacts of long non-coding RNAs (mESC RADICL-seq).

Table S2. Non-coding RNAs (mESC RADICL-seq) associated with chromatin structure only at the interchromosomal level. RNAs associated with more than 4 pairs of chromosomes are shown.

RNA	RNA type	Number of chromosome pairs
Snord49b	snoRNA	19
Mirg	lincRNA	12
Snord47	snoRNA	11
Lncpint	lincRNA	9
LSU-rRNA_Hsa_252	rRNA	9
1600020E01Rik	antisense	8
Gm50050	lincRNA	7
Pisd-ps2	transcribed_unprocessed_pseudogene	7
X_4_13396_c_mm10	ucaRNA	7
Gm27533	misc_RNA	6
Snhg1	processed_transcript	6
Snord43	miRNA	6
SSU-rRNA_Hsa_21	rRNA	6
B830012L14Rik	lincRNA	5
Dancr	processed_transcript	5
Zfas1	processed_transcript	5

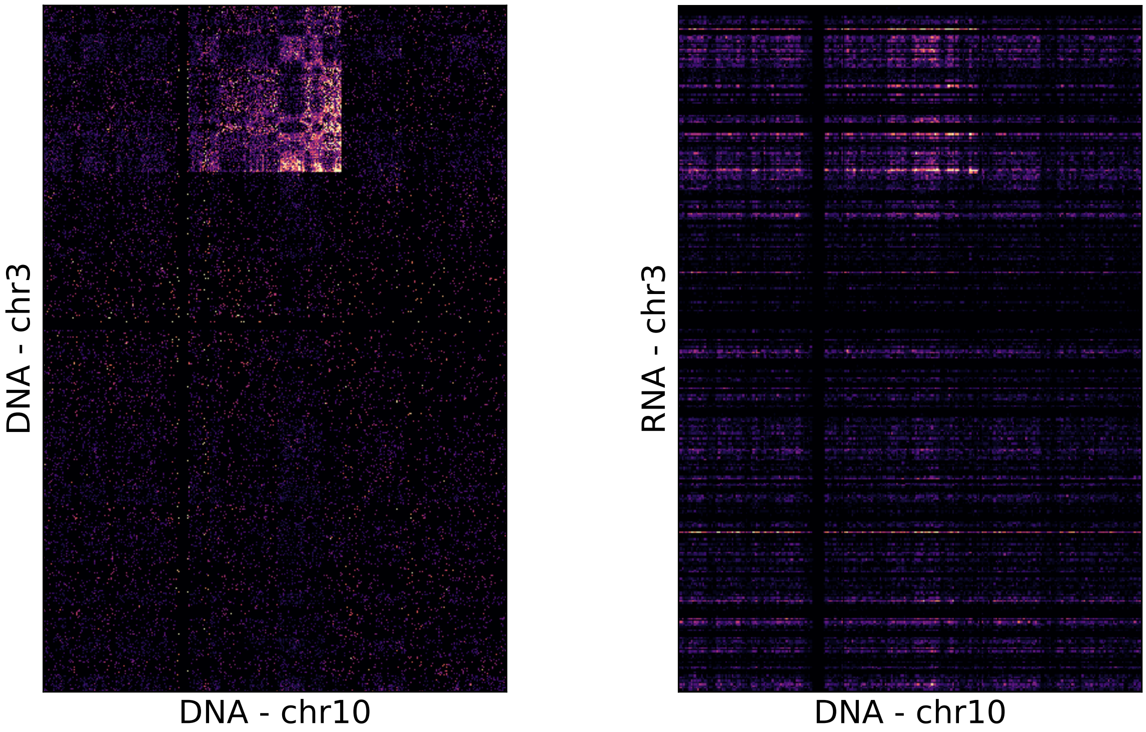

Figure S6. An example of the effect of chromosomal rearrangements of the K562 cell line on interactomes: Hi-C data on the left, Red-C data on the right. Brighter color corresponds to higher interactions frequency.

Table S3. The number of chromosomes (pairs of chromosomes for the interchromosomal case) on which there is an association of contacts with the chromatin structure (statistics were calculated on peaks counts, due to their small width and high coverage of "one-to-all" data).

RNA	RnaChromDB ID	Number of chromosomes	Number of chromosome pairs
Malat1	36	18	186
Malat1	52	20	178
Malat1	94	20	190
Firre	198	20	190
Xist	156	20	184

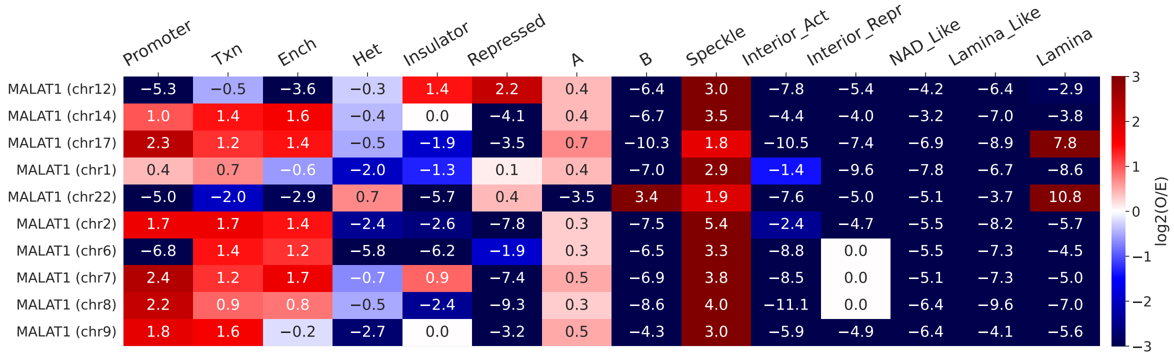

Figure S7. The enrichment of paired contacts of MALAT1 RNA in chromatin annotations on different chromosomes.

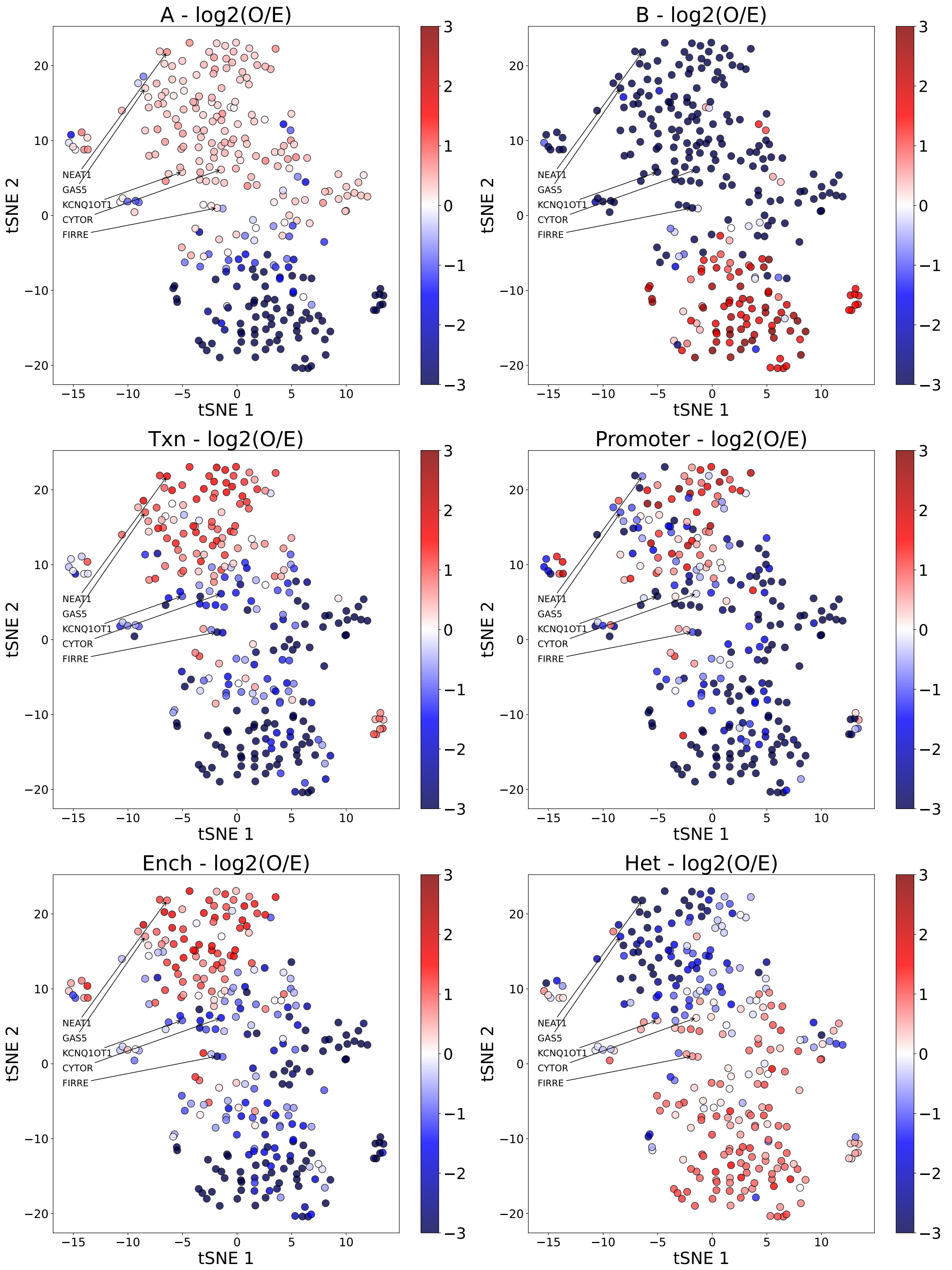

Figure S8. tSNE is colored by enrichment of paired contacts of non-coding RNAs in various chromatin state (continued on the next page).

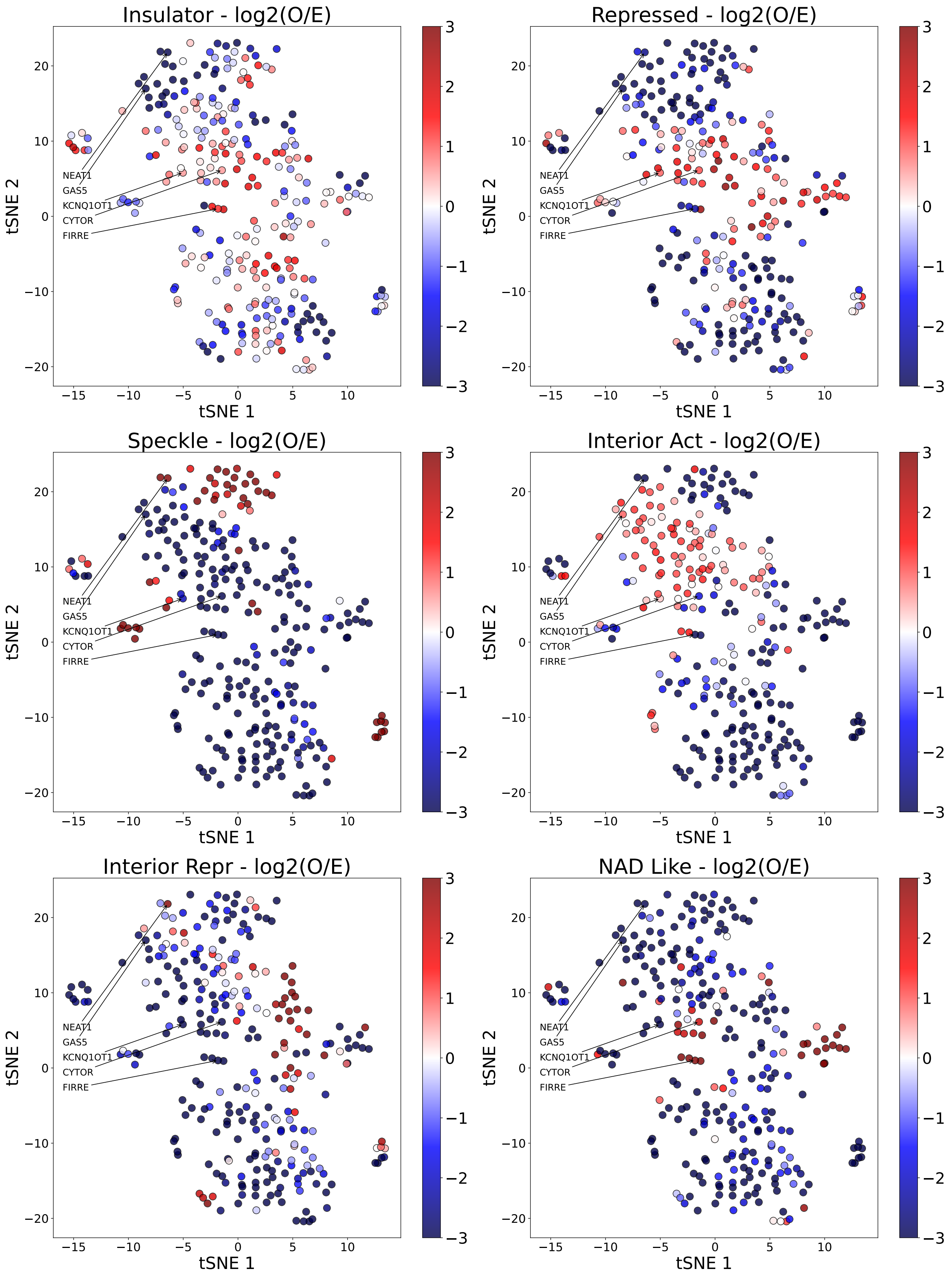

Figure S8. tSNE is colored by enrichment of paired contacts of non-coding RNAs in various chromatin state (continued on the next page).

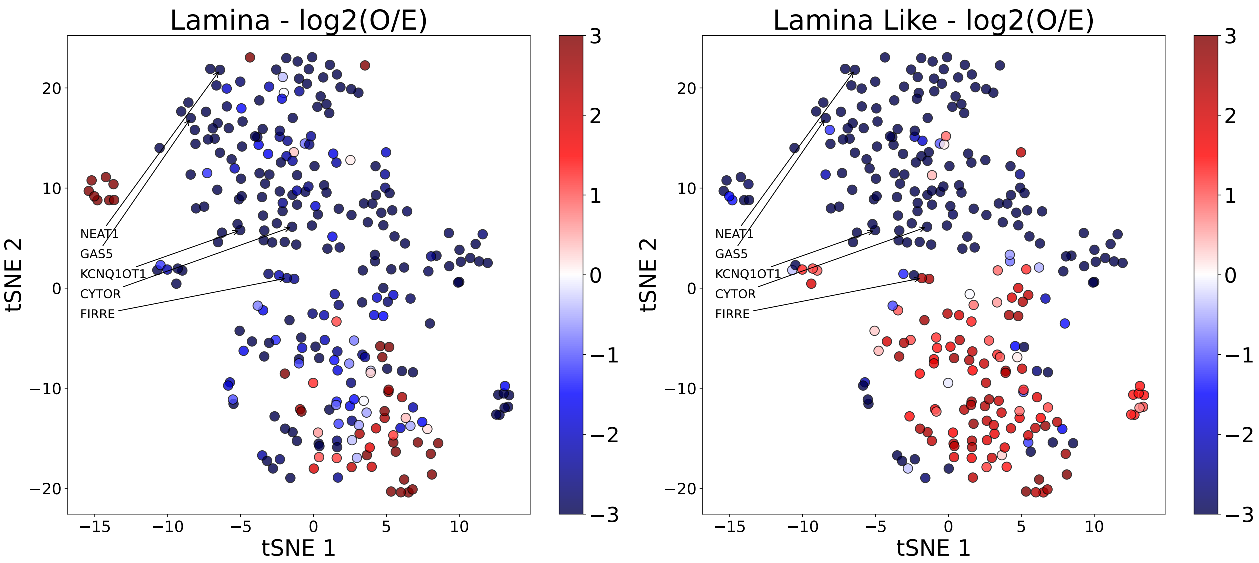

Figure S8. tSNE is colored by enrichment of paired contacts of non-coding RNAs in various chromatin state

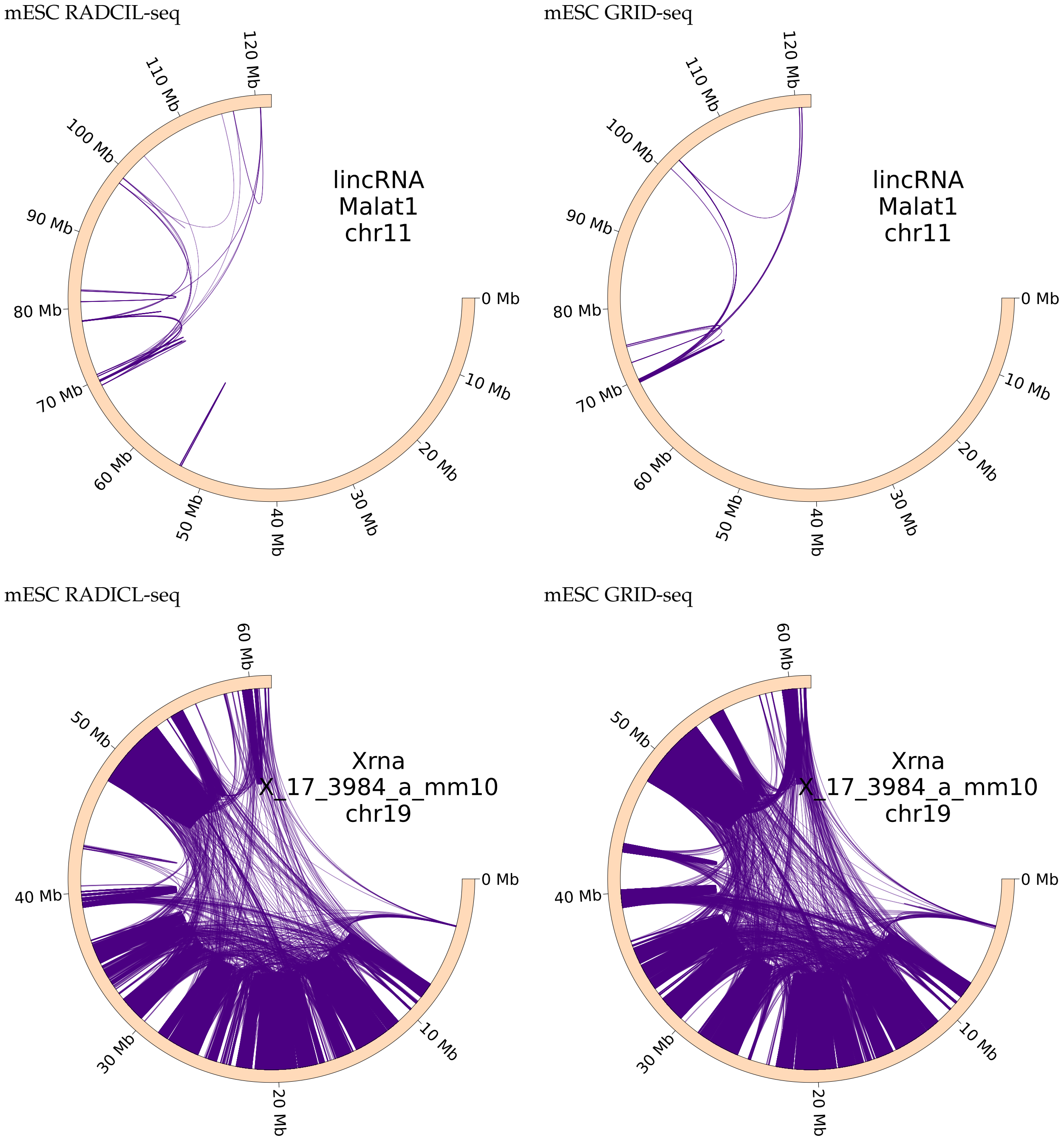

Figure S9. Examples of paired contacts of RNA from the protocols RADICL-seq (left) and GRID-set (right) of the mESC cell line. The top row is Malat1. The bottom row is X_17_3984_a_mm10.

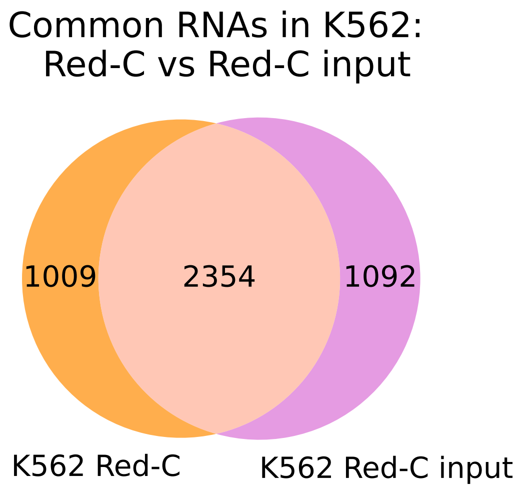

Figure S10. The intersection of sets of RNAs associated with the chromatin structure between the Red-C and Red-C (input) protocols, cell line K562, on the left - all RNAs, on the right - non-coding RNAs.

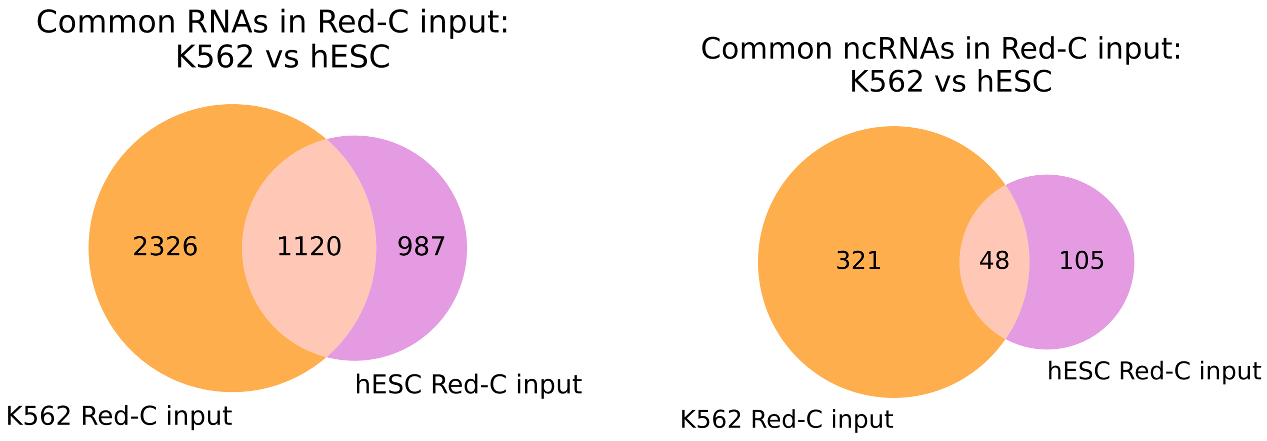

Figure S11. The intersection of the sets of RNAs associated with the chromatin structure between the protocols of the K562 and hESC cell lines, the Red-C (input) protocol, on the left - all RNAs, on the right - non-coding RNAs.

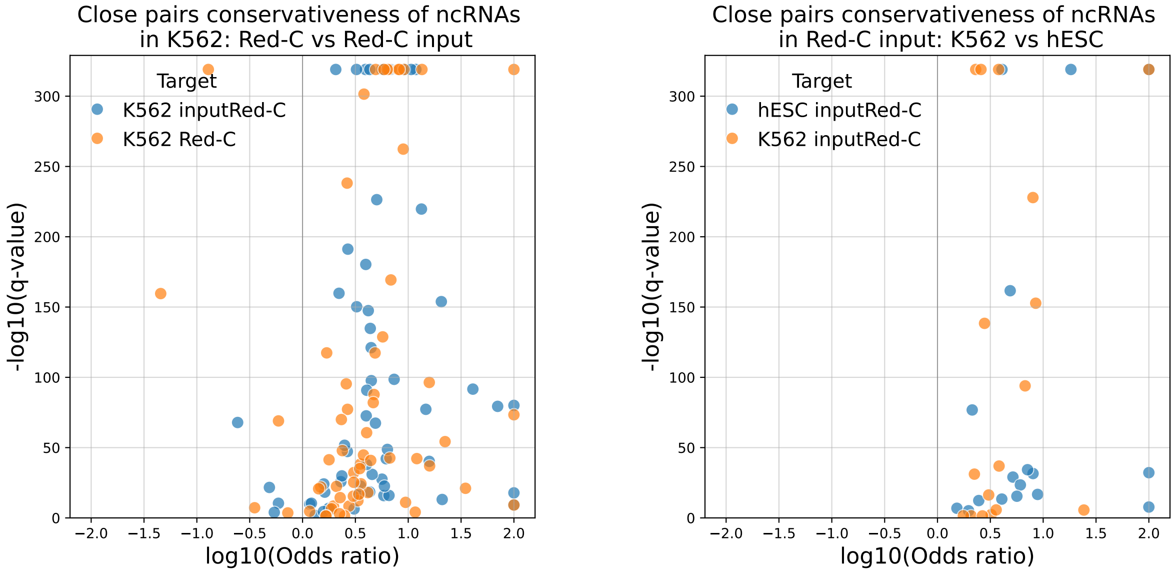

Figure S12. The relationship of the q-values and the odds ratios of the Fisher's exact test. On the left - comparison of Red-C and Red-C (input) experiments, on the right comparison of K562 and hESC cell lines.

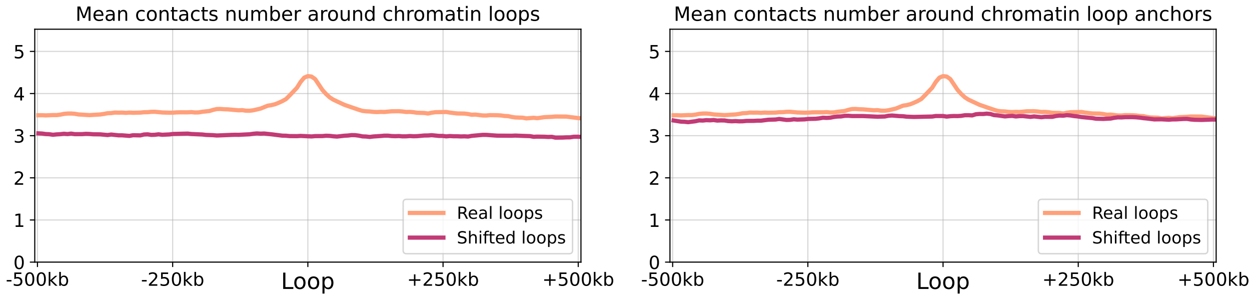

Figure S13. The average number of RNA-DNA (K562 Red-C) interactions in the vicinity of chromatin loop anchors, on the left the background model is random shift, on the right the background model is shift, taking into account belonging to the A/B compartment.

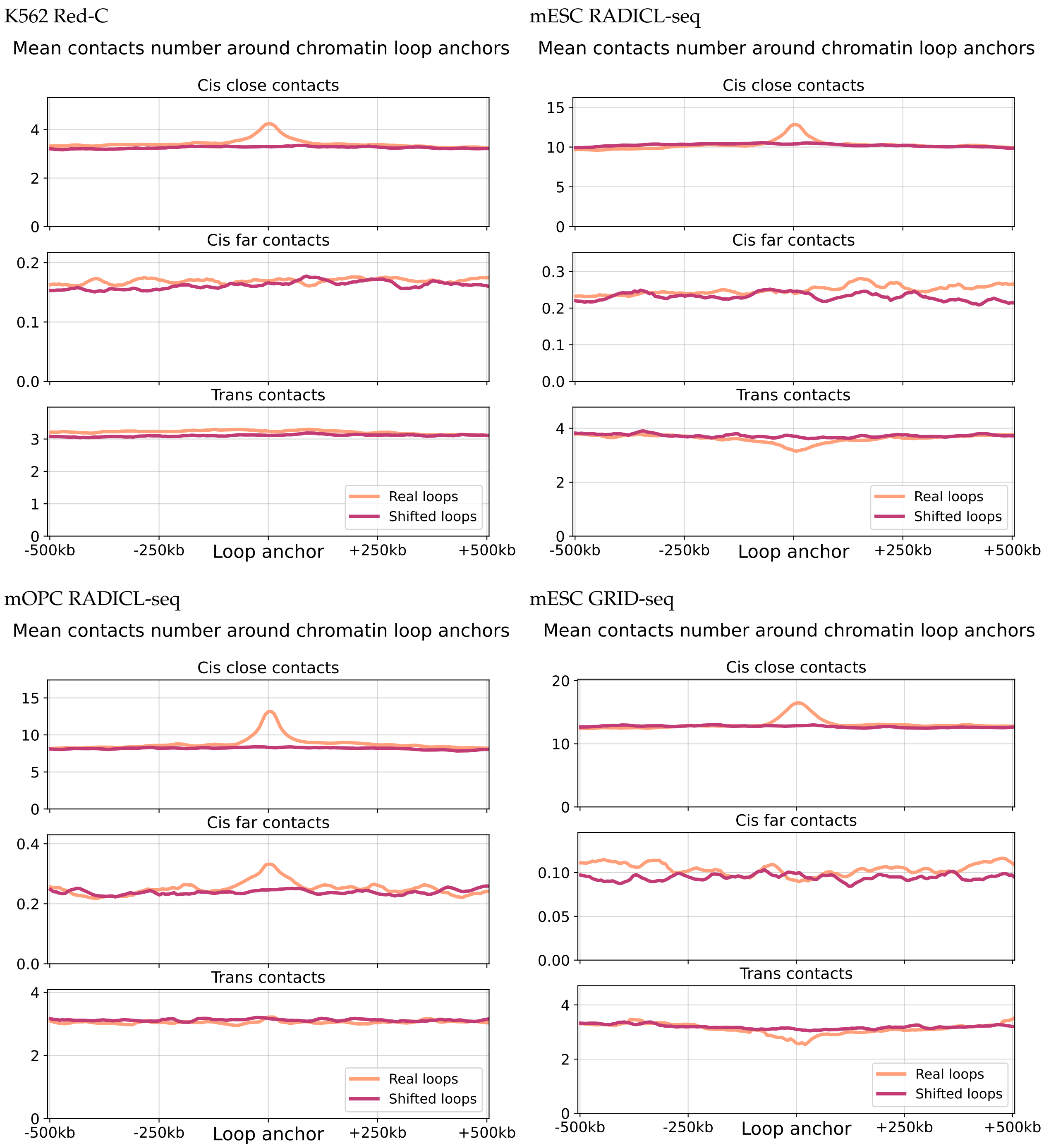

Figure S14. The average number of RNA-DNA interactions in peaks in the vicinity of chromatin loop anchors (continued on the next page).

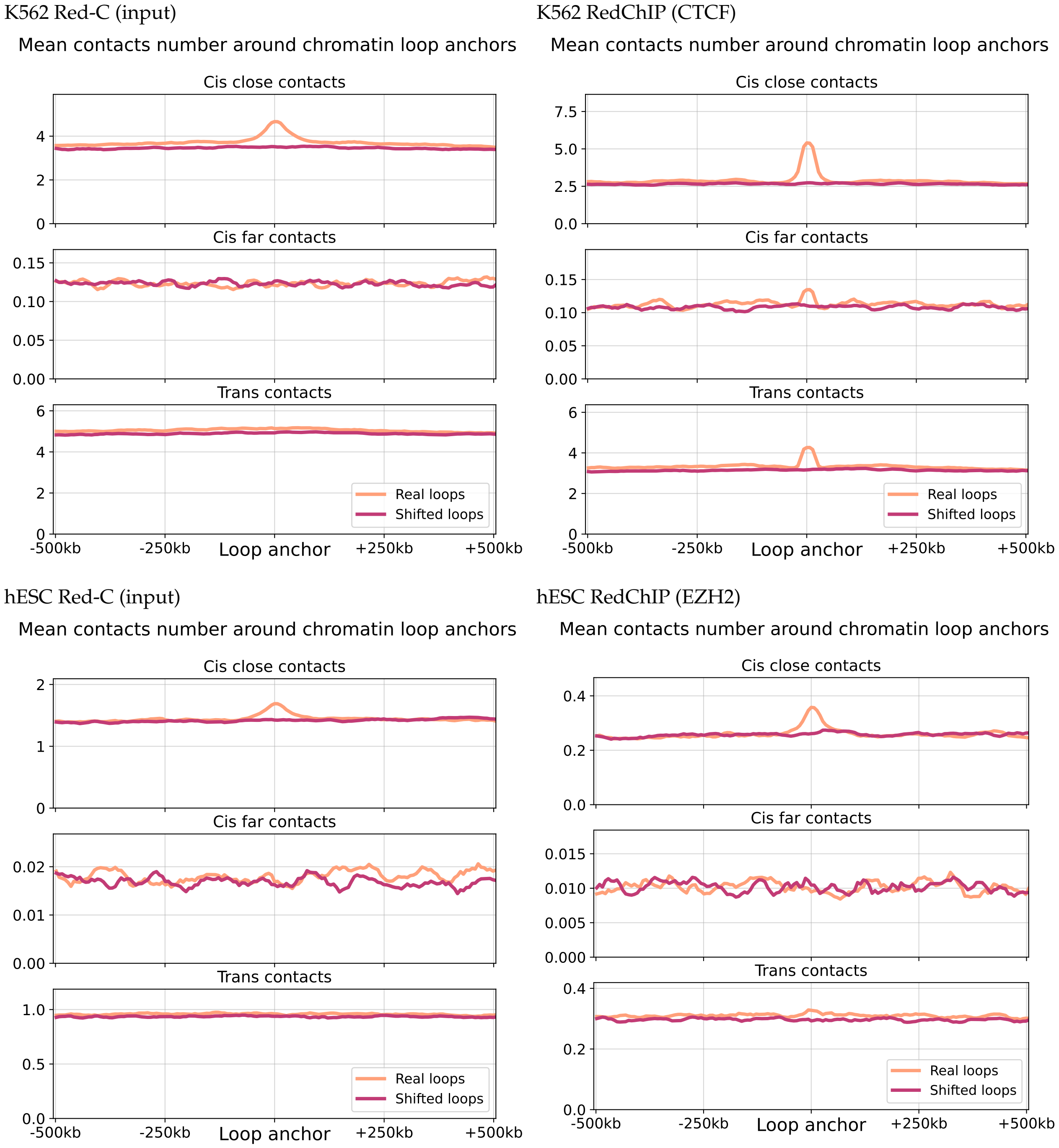

Figure S14. The average number of RNA-DNA interactions in peaks in the vicinity of chromatin loops anchors.

K562 Red-C, full set of RNA-DNA contacts

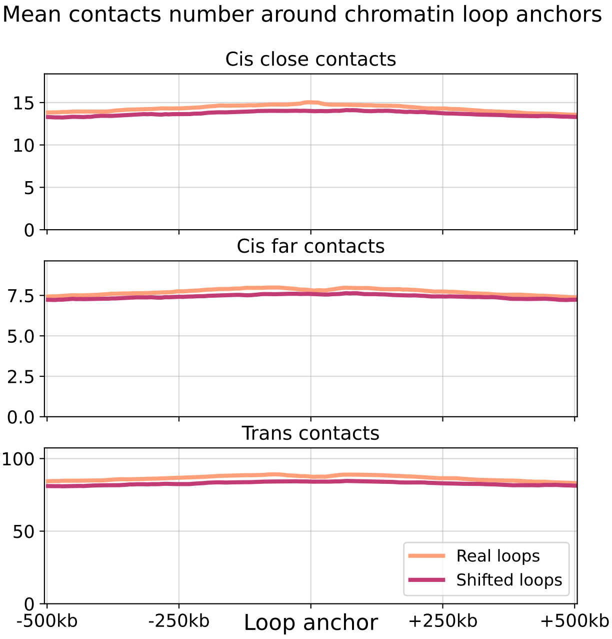

mESC RADICL-seq, full set of RNA-DNA contacts

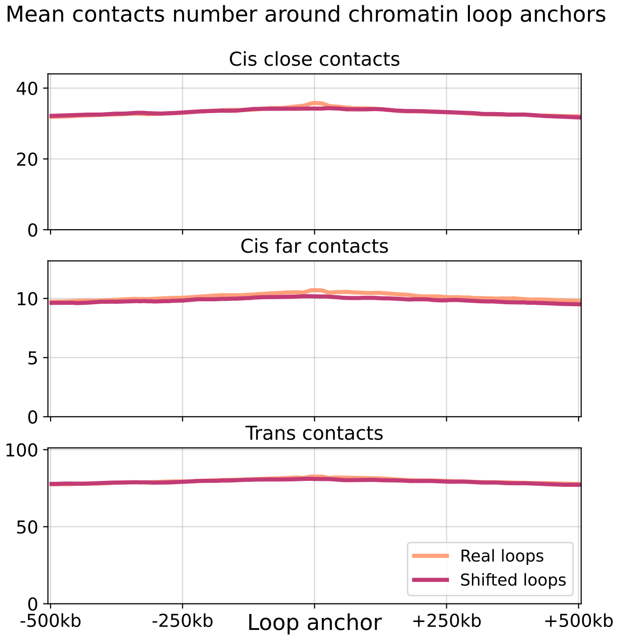

mOPC RADICL-seq, full set of RNA-DNA contacts

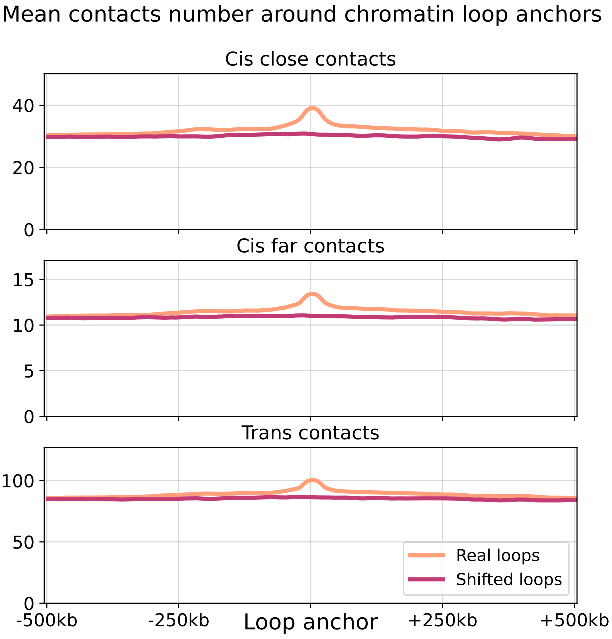

mESC GRID-seq, full set of RNA-DNA contacts

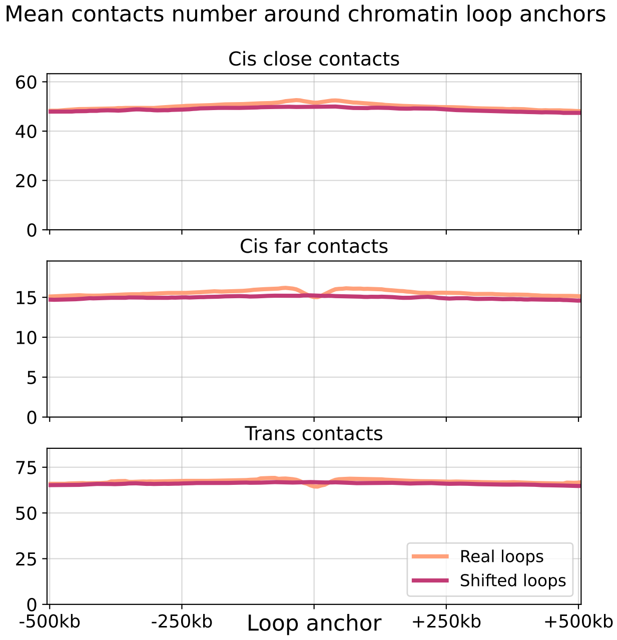

Figure S15. The average number of the whole set of RNA-DNA interactions in the vicinity of chromatin loop anchors (continued on the next page).

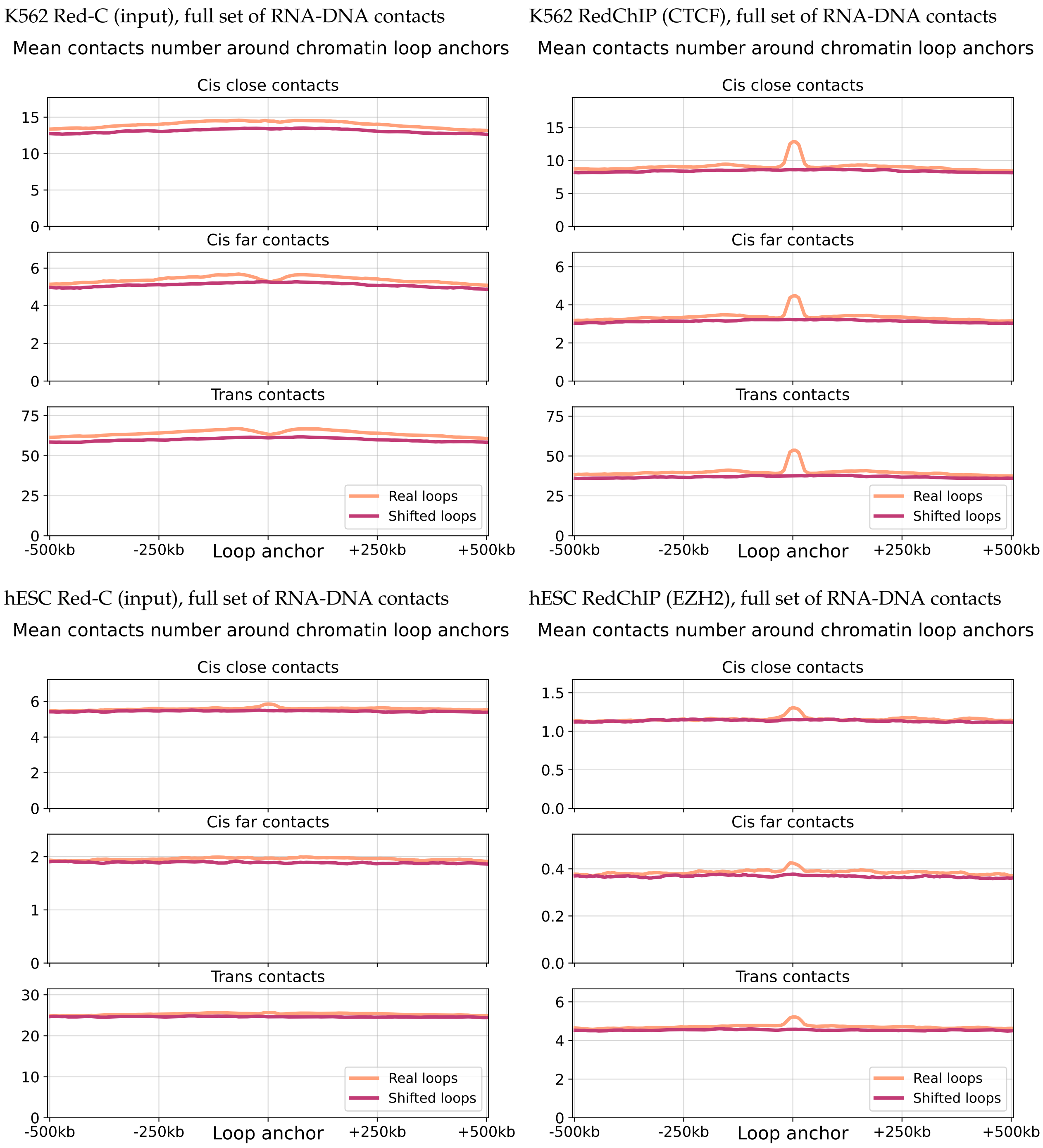

Figure S15. The average number of the whole set of RNA-DNA interactions in the vicinity of chromatin loops anchors.

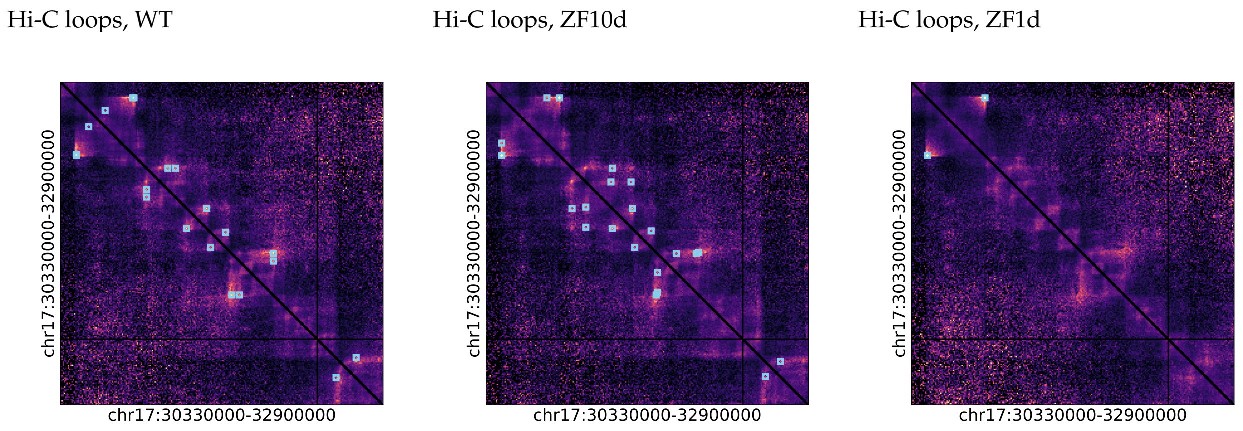

Figure S16. Examples of annotated loops on Hi-C maps in different conditions, on the left - WT, in the center - deletion in ZF10, on the right - deletion in ZF1 (chr17:30330000:32900000). Brighter color corresponds to higher interactions frequency.

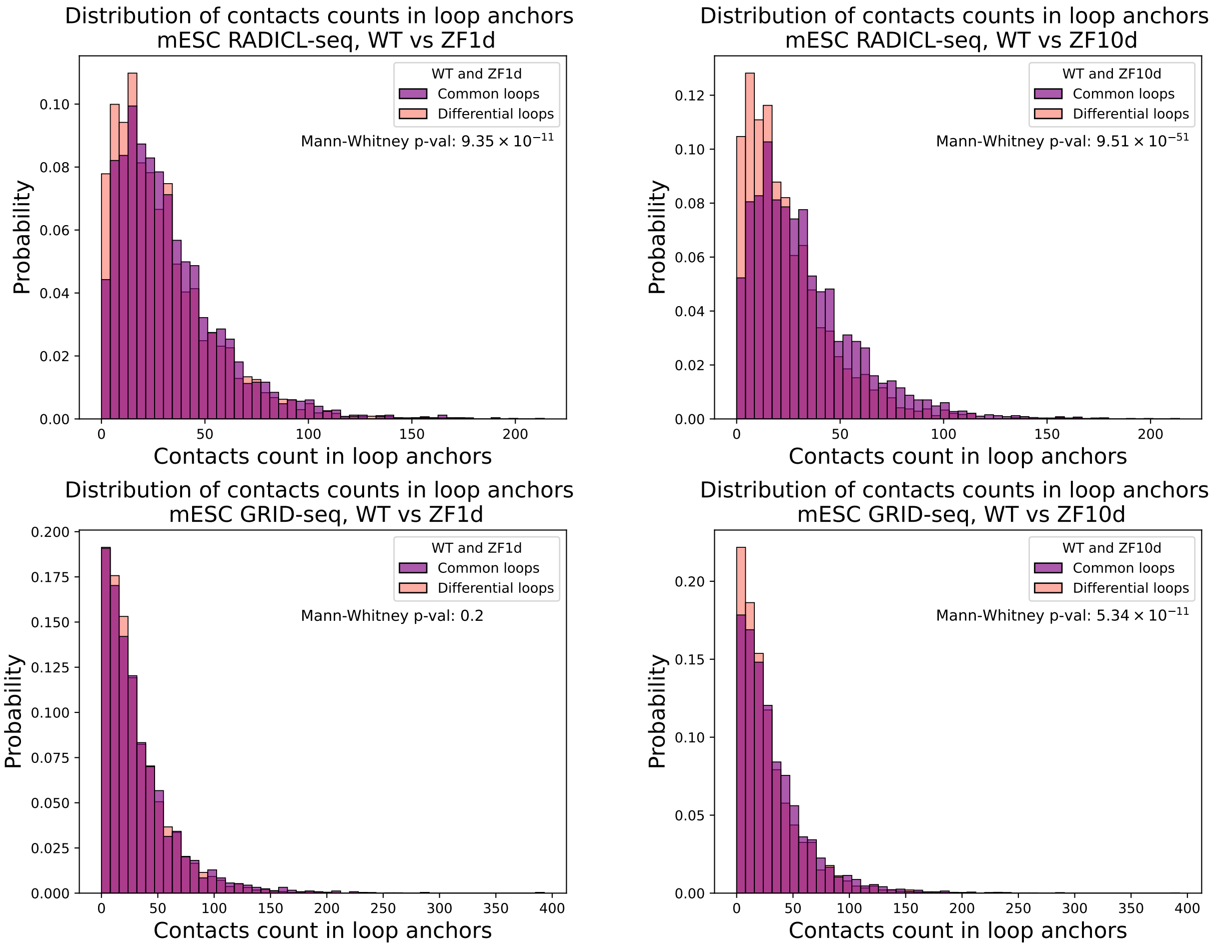

Figure S17. Comparison of the distribution of the number of RD-contacts in mESC within the anchors of chromatin loops. Common loops are found in both WT and mutant lines. Differential loops are absent in the mutant cell line (on the left is a deletion in the ZF1 domain, on the right is in the ZF10 domain, the upper row is RADICL-seq, the lower one is GRID-seq).

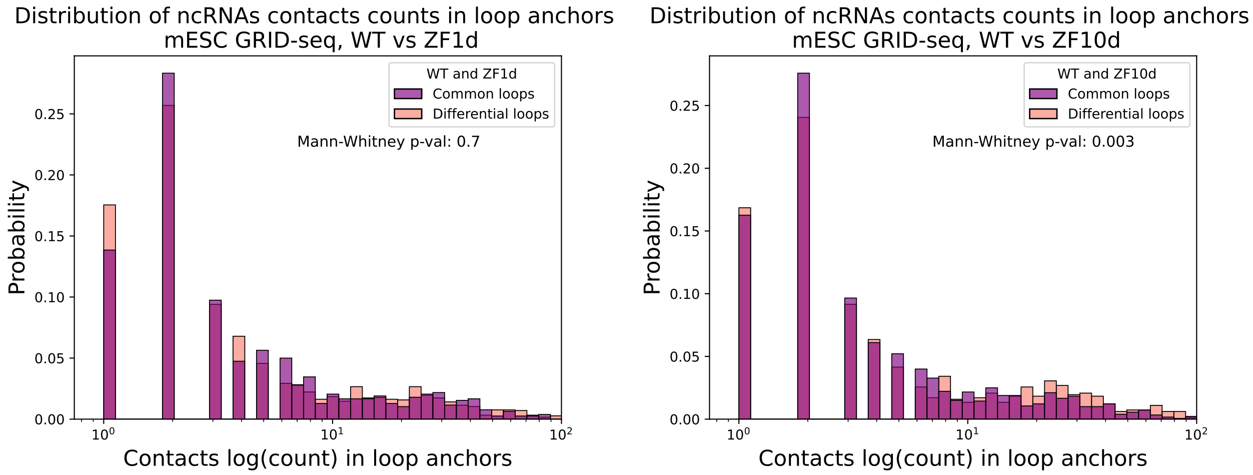

Figure S18. Comparison of the distribution of the number of ncRNAs RD-contacts in mESC GRID-seq within the anchors of chromatin loops. Common loops are found in both WT and mutant lines. Differential loops are absent in the mutant cell line (on the left is a deletion in the ZF1 domain, on the right is in the ZF10 domain.

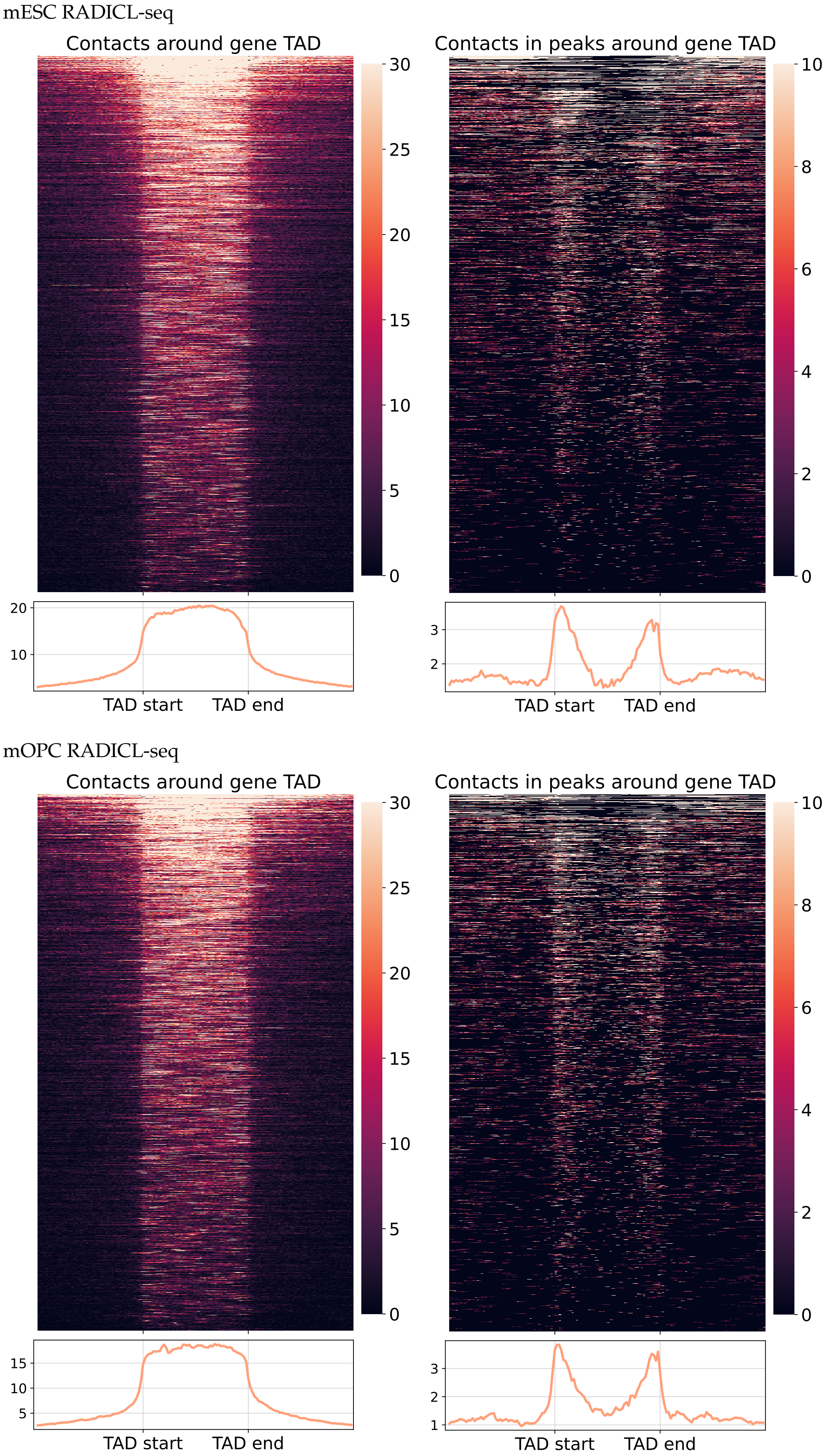

Figure S19. Left - distribution of the full set of RD-contacts around the TADs of RNAs genes (Red-C K562), right - distribution of RD-contacts from BaRDIC peaks around the TADs of RNAs genes (continued on the next page).

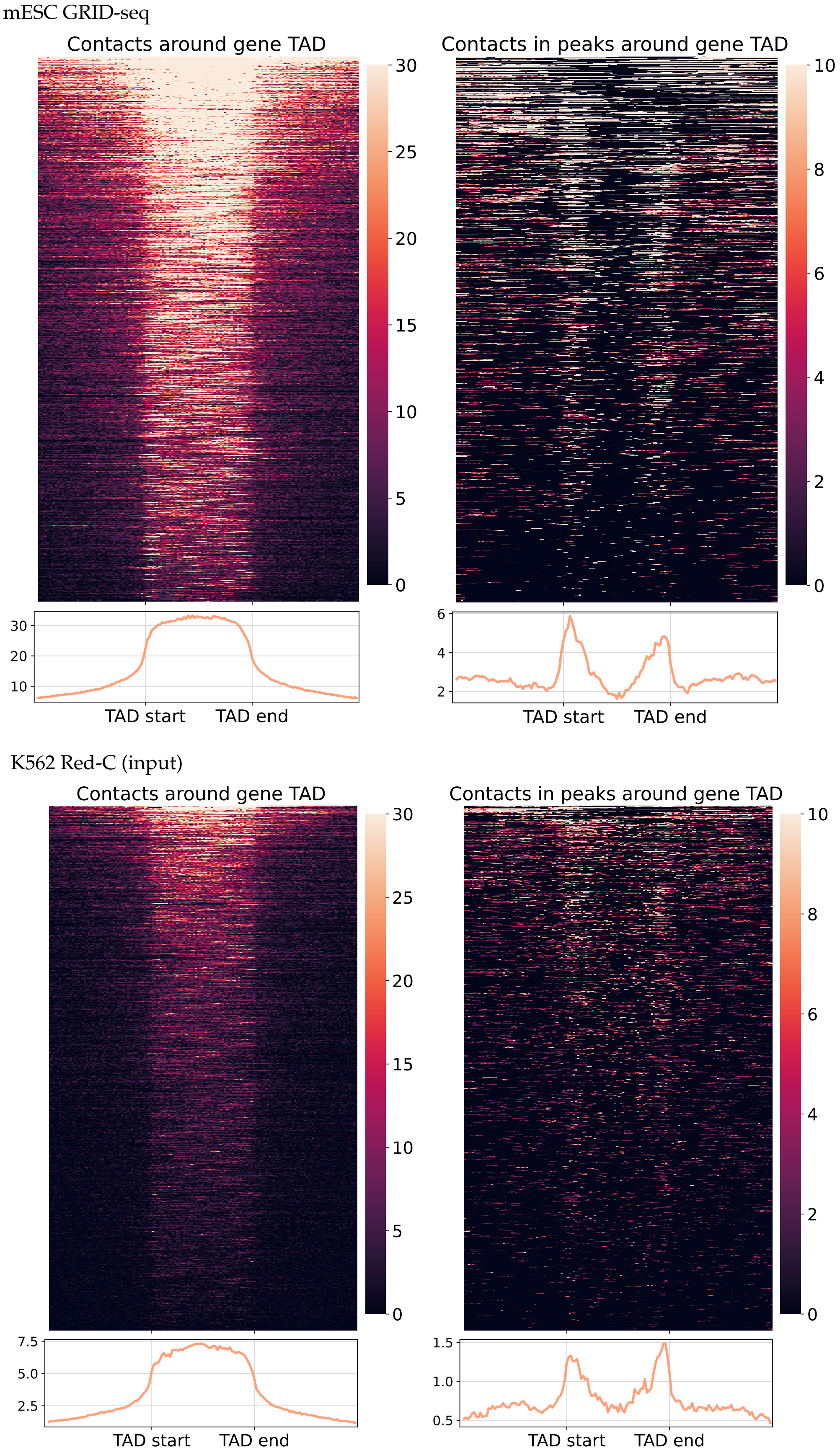

Figure S19. Left - distribution of the full set of RD-contacts around the TADs of RNAs genes (Red-C K562), right - distribution of RD-contacts from BaRDIC peaks around the TADs of RNAs genes (continued on the next page).

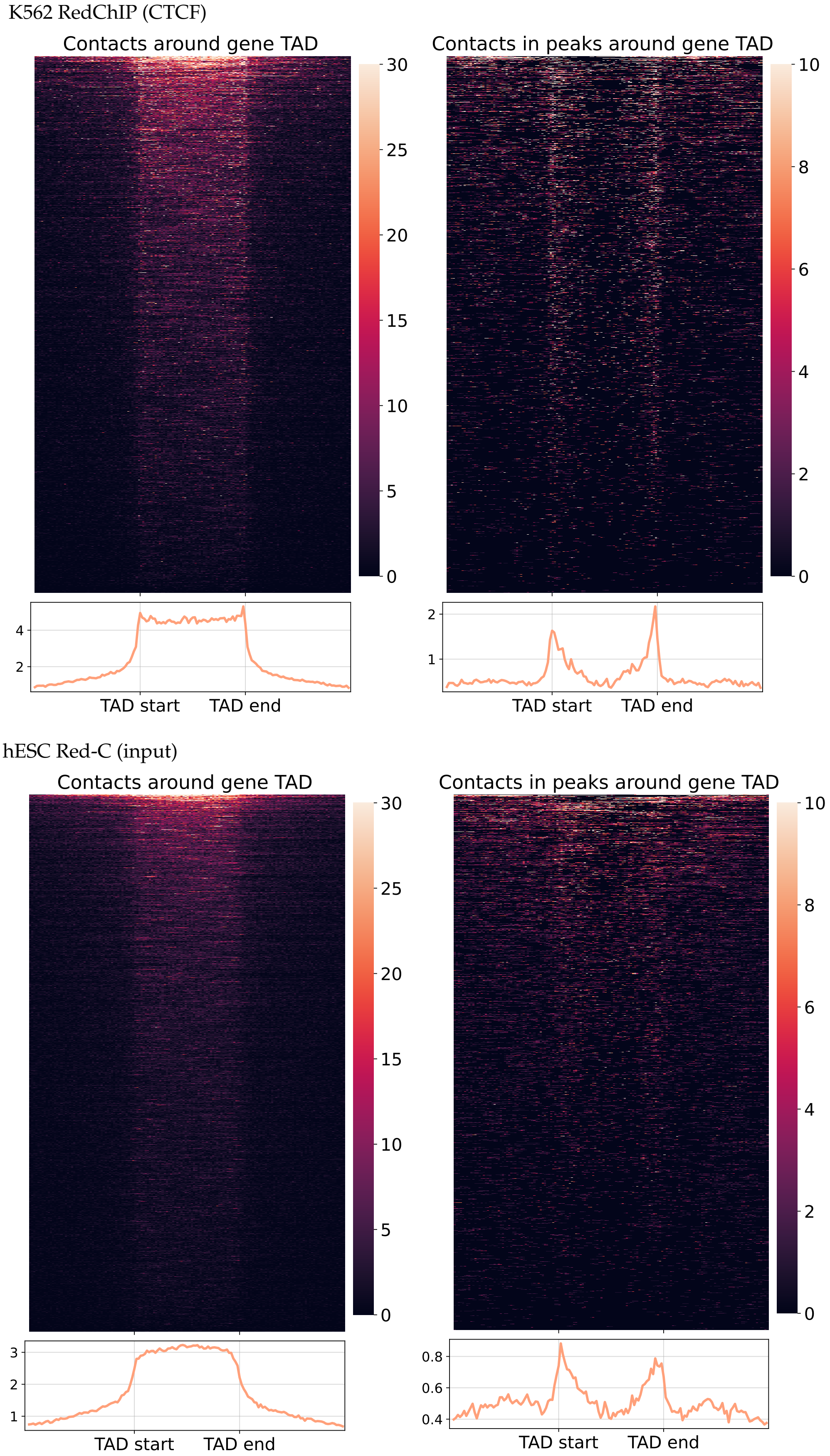

Figure S19. Left - distribution of the full set of RD-contacts around the TADs of RNAs genes (Red-C K562), right - distribution of RD-contacts from BaRDIC peaks around the TADs of RNAs genes (continued on the next page).

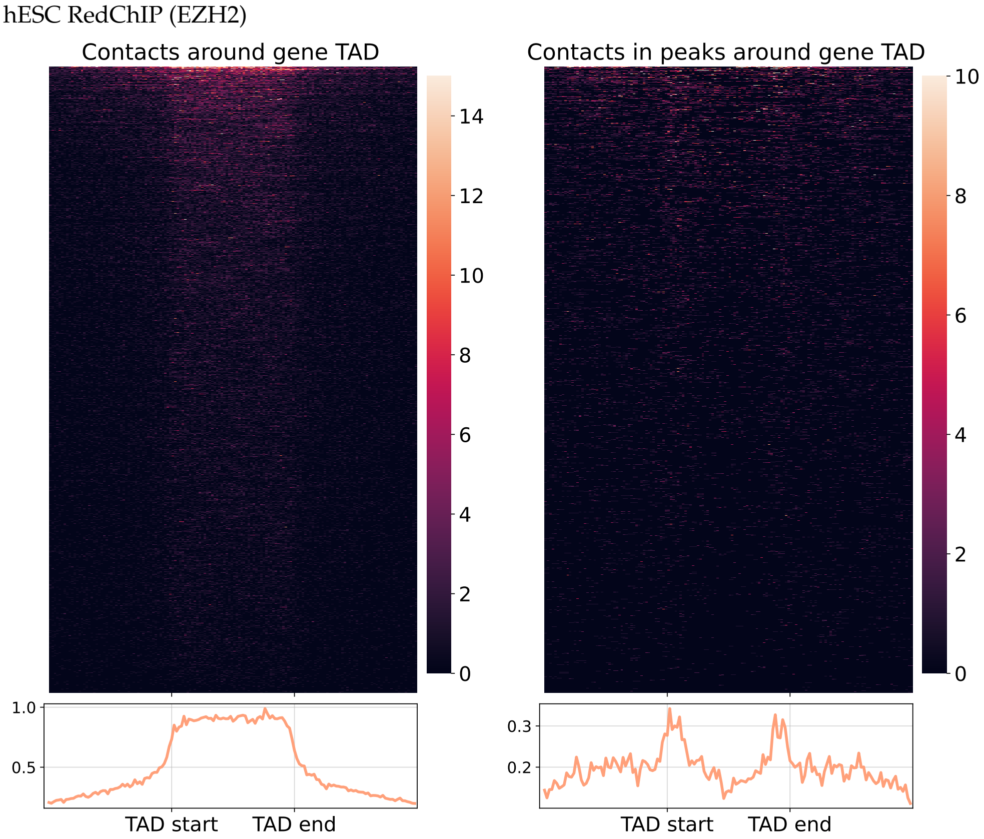

Figure S19. Left - distribution of the full set of RD-contacts around the TADs of RNAs genes (Red-C K562), right - distribution of RD-contacts from BaRDIC peaks around the TADs of RNAs genes.

Table S4. A two-sided Mann-Whitney test for OutInDR values of different RNA biotypes and mRNA (NS - non significant, adj p-value > 0.05).

Cell line	RNA type	Mann-Whitney p-value (Bonferroni correction)
K562 Red-C	lncRNA	3.7×10^{-8}
	vlincRNA	2.23×10^{-21}
	pseudogene	NS
	ucaRNA	NS
K562 Red-C (input)	lncRNA	9.9×10^{-7}
	vlincRNA	8.46×10^{-11}
	pseudogene	NS
	ucaRNA	NS
hESC Red-C (input)	lncRNA	NS
	vlincRNA	NS
	pseudogene	NS
	ucaRNA	NS
mESC RADICL-seq	lincRNA	NS
	antisense	NS
	processed transcript	NS
	ucaRNA	4.24×10^{-9}
	TEC	NS
mOPC RADICL-seq	lincRNA	NS
	antisense	NS
	processed transcript	NS
	ucaRNA	2.6×10^{-13}
	TEC	NS
mESC GRID-seq	lincRNA	0.032
	antisense	NS
	processed transcript	NS
	ucaRNA	7.33×10^{-11}
	TEC	2.6×10^{-8}

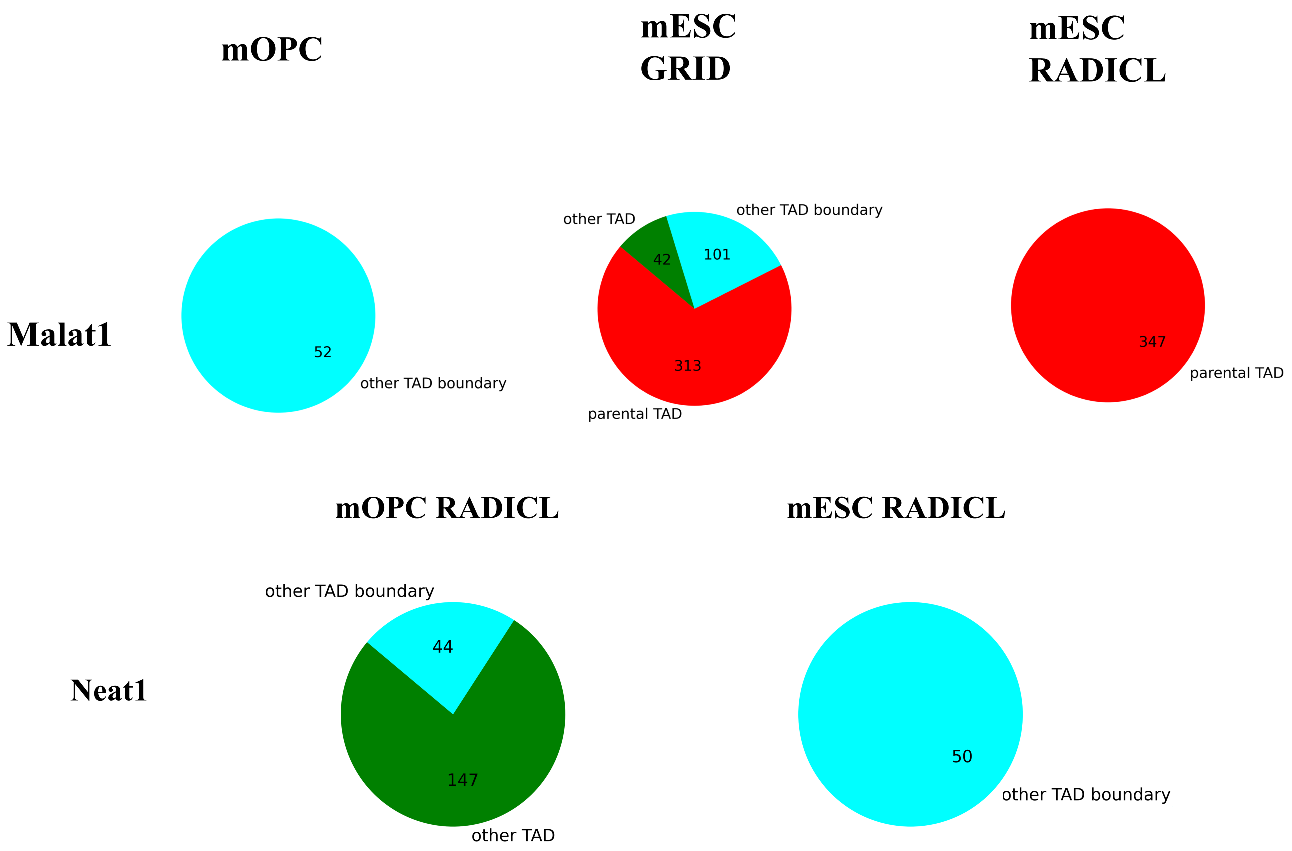

Figure S20. RD-contacts distribution in TADs and boundaries in different cell lines. Top row - MALAT1 contacts, bottom row - NEAT1.

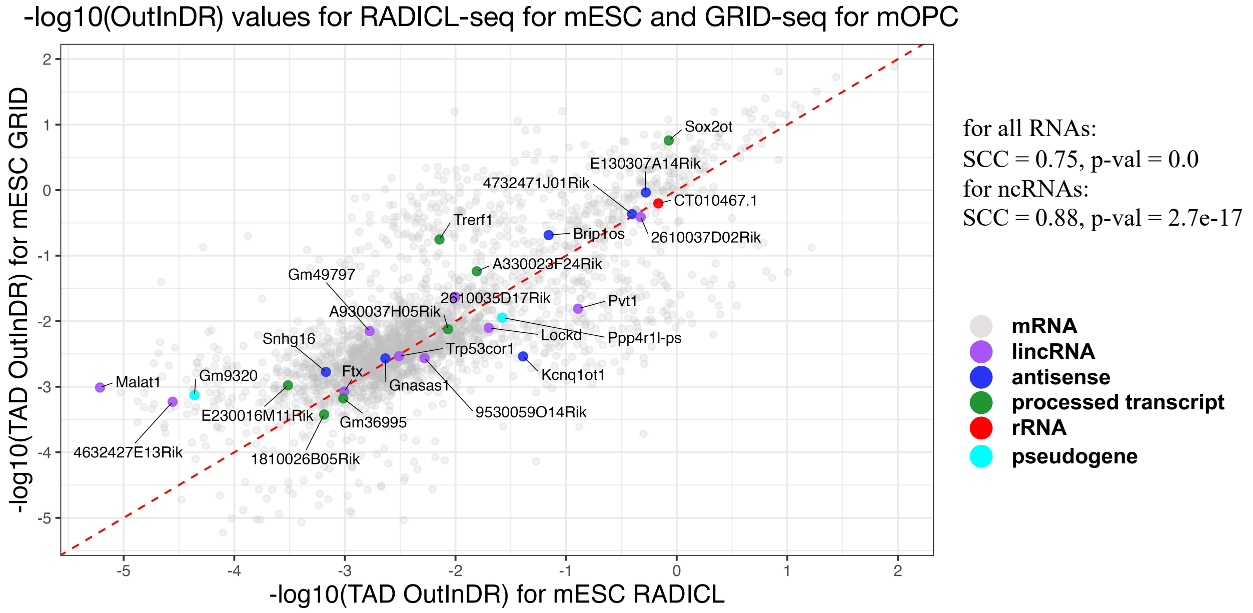

Figure S21. Scatterplot with values -log10(OutInDR) for the RADICL-seq experiment on the X axis and GRID-seq on the Y axis for the mESC cell line.

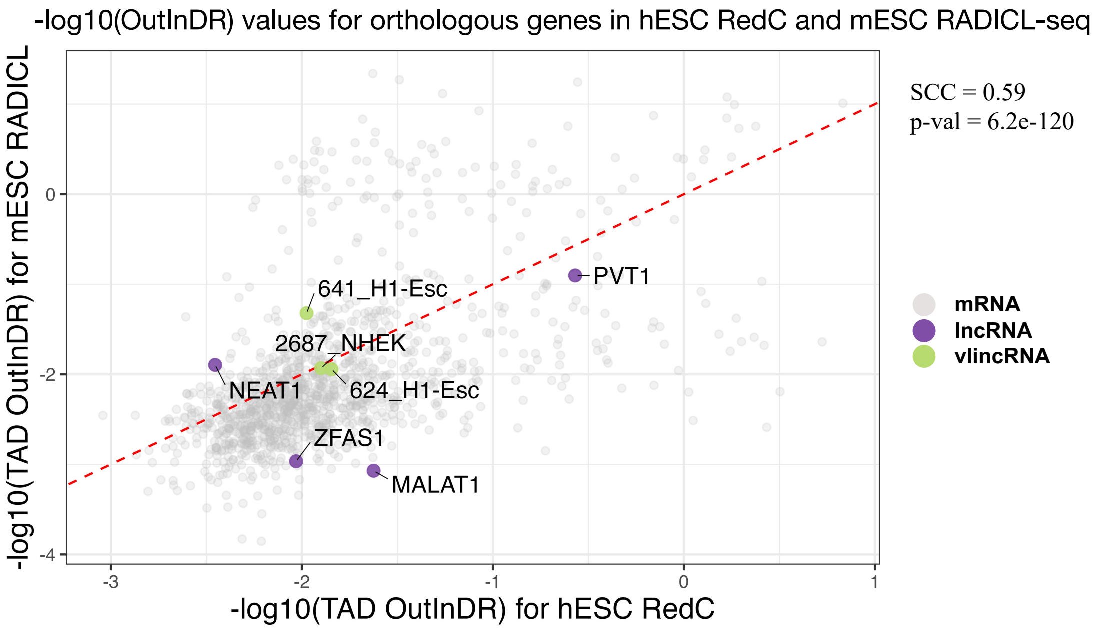

Figure S22. Scatterplot with values -log10(OutInDR) of orthologous genes in the hESC (X-axis) and mESC RADICL-seq (Y-axis) cell lines.

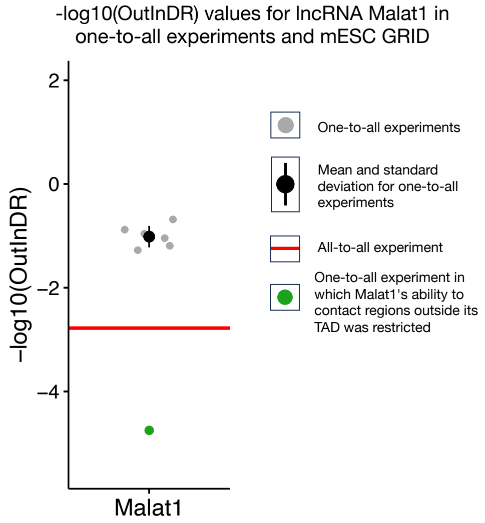

Figure S23. Comparison of the -log10(OutInDR) value for lincRNA Malat1 in "one-to-all" and "all-to-all" experiments for mESC GRID-seq.

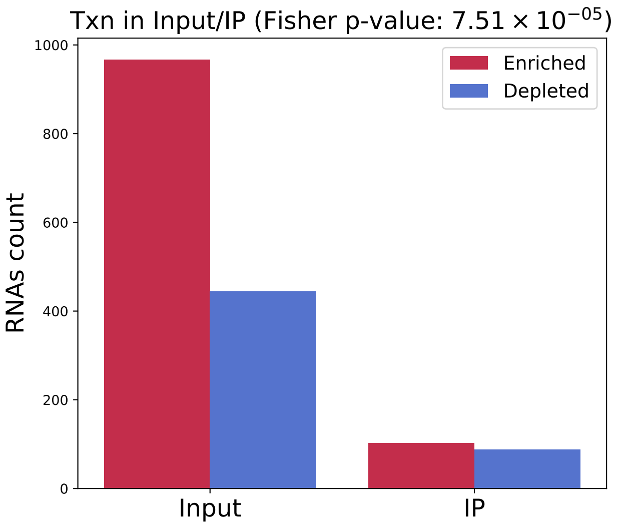

Figure S24. The number of cases of significant overrepresentation and underrepresentation of paired contacts in pairs of the active transcription state in Red-C and RedChIP EZH2 (hESC).

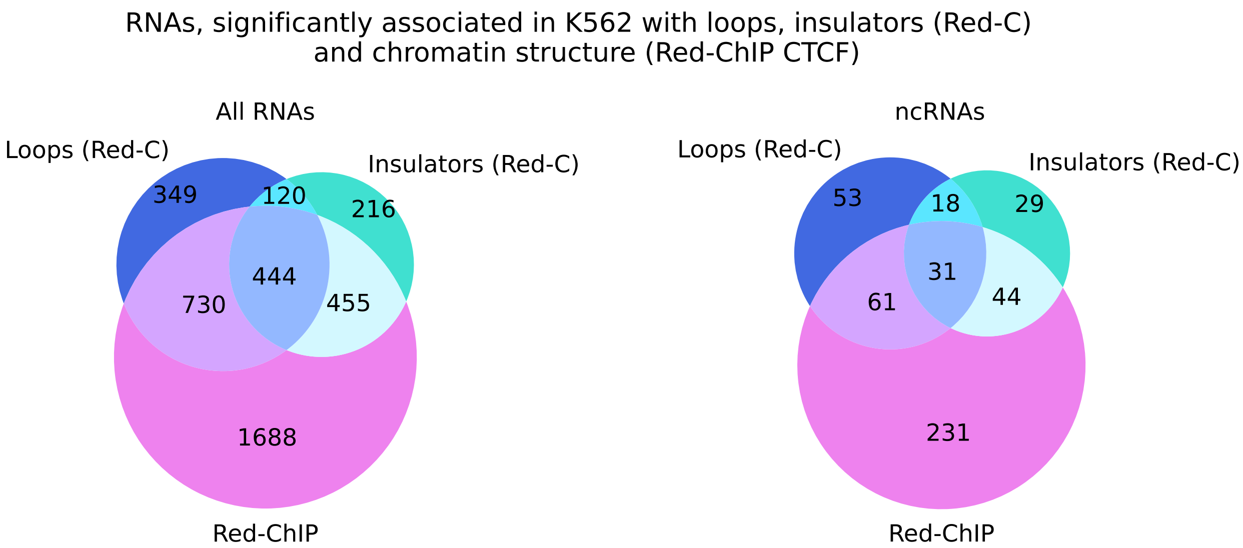

Figure S25. The intersection of RNAs which are significantly associated with loops (Red-C data), with the chromatin structure (Red-ChIP), and whose paired contacts are associated with insulators (Red-C).

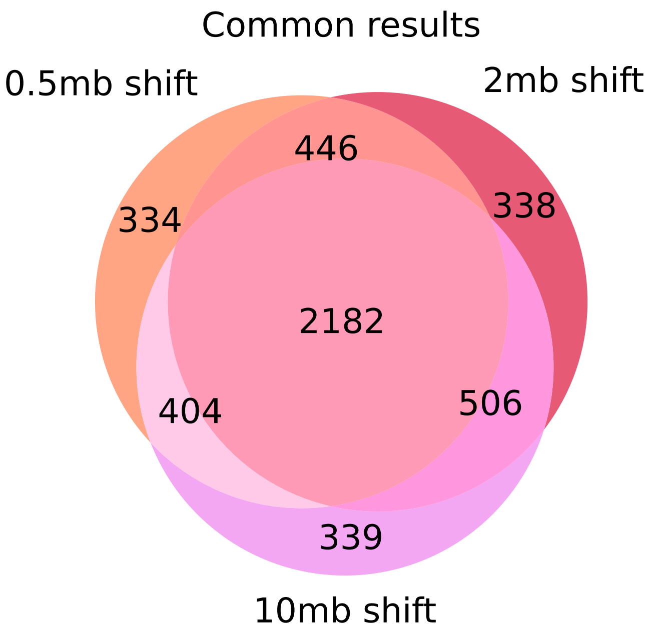

Figure S26. The intersection of the results of significant association of RNA-DNA interactions and chromatin structure depending on the shift (K562 Red-C). The numbers of different results are close between the shift values, which indicates that the magnitude of the shift does not affect the degree of difference between the real model and the background one.

Table S5. ChromHMM states grouping [1].

**Grouped state**	**ChromHMM state**
Promoter	1_Active_Promoter<br>2_Weak_Promoter<br>3_Poised_Promoter
Ench	4_Strong_Enhancer<br>5_Strong_Enhancer<br>6_Weak_Enhancer<br>7_Weak_Enhancer
Insulator	8_Insulator
Txn	9_Txn_Transition<br>10_Txn_Elongation<br>11_Weak_Txn
Repressed	12_Repressed
Het	13_Heterochrom/lo<br>14_Repetitive/CNV<br>15_Repetitive/CNV

Table S6. SPIN states grouping [2].

**Grouped state**	**SPIN state**
Speckles	Speckles
Interior_Act	Interior_Act1<br>Interior_Act2<br>Interior_Act3
Interior_Repr	Interior_Repr1
NAD_like	Interior_Repr2
Lamina_Like	Lamina_Like<br>Near_Lm1<br>Near_Lm2
Lamina	Lamina

References

1. Ernst, J.; Kheradpour, P.; Mikkelsen, T.S.; Shoresh, N.; Ward, L.D.; Epstein, C.B.; Zhang, X.; Wang, L.; Issner, R.; Coyne, M.; et al. Mapping and analysis of chromatin state dynamics in nine human cell types. *Nature* **2011**, *473*, 43–49. Number: 7345, https://doi.org/10.1038/nature09906.
2. Wang, Y.; Zhang, Y.; Zhang, R.; van Schaik, T.; Zhang, L.; Sasaki, T.; Peric-Hupkes, D.; Chen, Y.; Gilbert, D.M.; van Steensel, B.; et al. SPIN reveals genome-wide landscape of nuclear compartmentalization. *Genome Biology* **2021**, *22*, 36. Number: 1, https://doi.org/10.1186/s13059-020-02253-3.
3. Gavrilov, A.A.; Zharikova, A.A.; Galitsyna, A.A.; Luzhin, A.V.; Rubanova, N.M.; Golov, A.K.; Petrova, N.V.; Logacheva, M.D.; Kantidze, O.L.; Ulianov, S.V.; et al. Studying RNA–DNA interactome by Red-C identifies noncoding RNAs associated with various chromatin types and reveals transcription dynamics. *Nucleic Acids Research* **2020**, *48*, 6699–6714. Number: 12 Publisher: Oxford Academic, https://doi.org/10.1093/nar/gkaa457.
4. Gavrilov, A.A.; Sultanov, R.I.; Magnitov, M.D.; Galitsyna, A.A.; Dashinimaev, E.B.; Aiden, E.L.; Razin, S.V. Brief Report: RedChIP identifies noncoding RNAs associated with genomic sites occupied by Polycomb and CTCF proteins. *Proceedings of the National Academy of Sciences of the United States of America* **2022**, *119*. Publisher: National Academy of Sciences, https://doi.org/10.1073/pnas.2116222119.
5. Bonetti, A.; Agostini, F.; Suzuki, A.M.; Hashimoto, K.; Pascarella, G.; Gimenez, J.; Roos, L.; Nash, A.J.; Ghilotti, M.; Cameron, C.J. et al. RADICL-seq identifies general and cell type–specific principles of genome-wide RNA-chromatin interactions. *Nature Communications* **2020**, *11*, 1018. https://doi.org/10.1038/s41467-020-14337-6.
6. Li, X.; Zhou, B.; Chen, L.; Gou, L.T.; Li, H.; Fu, X.D. GRID-seq reveals the global RNA–chromatin interactome. *Nature Biotechnology* **2017**, *35*, 940–950. https://doi.org/10.1038/nbt.3968.
