# Supplementary material for: A Joint Analysis of RNA-DNA and DNA-DNA Interactomes Reveals Their Strong Association
Source: Int J Mol Sci. 2025 Jan 28;26(3):1137. doi: 10.3390/ijms26031137 (PMC11817408; doi:10.3390/ijms26031137)
Supplement: Supplementary file 1 [file ijms-26-01137-s001.zip › rnadna_hic_paper_supplementary_note.pdf]

Supplementary Materials: Joint analysis of RNA-DNA and DNA-DNA interactomes reveals their strong association

Supplementary note S1

For K562 cells, there are many instances of RNA associations with chromatin structure at the interchromosomal level (for 450 out of 6000 RNAs). However, this cell line is characterized by chromosomal rearrangements that distort both Hi-C and RNA-chromatin interactions. Many observed interchromosomal contacts and associated RNA pairs are, in fact, intrachromosomal between parts of fused chromosomes (examples on Supplementary Figure S6). This distorts the analysis, so we did not interpret the results on the K562 line.

Supplementary note S2

In addition to consistent cases of functional annotations states enrichment, we also observe a number of contradictory results. For example, on chromosomes 12, 17 and 22, the paired contacts of MALAT1 (Supplementary Figure S7) are enriched in lamina and repressed chromatin. Also, for NEAT1, the association of paired contacts in the inactive chromatin of the SPIN annotation is observed. These inconsistent cases are in the minority and are most likely due to imperfect annotations and the noisiness of RNA-chromatin interactions data, which was not completely eliminated by the BaRDIC tool. In addition, these RNAs are highly expressed [1,2] and only a small proportion of these molecules is involved in the formation of speckles and paraspeckles.

We analyzed less or completely unexplored, but highly contacting RNAs for the interpretation of their possible functions based on the enrichment of paired contacts within chromatin states. We selected examples with observed-to-expected ratio > 2, adjusted p-value (chi-square) < 0.05 and the number of paired contacts on the chromosome > 1000 (the full table is given in the Supplementary Data). For promoters, we considered the very long non-coding RNA (vlincRNA) 1935_HUVEC (chr2), lncRNA AL590666.2 (chr1), ucaRNA X_11_61_b_hg38 (chr11). In addition to promoters, all of these RNAs prefer different states of active chromatin and avoid repressed or heterochromatin. One can assume that they are involved in the activation of transcription of a number of genes or in other processes within active chromatin. Examples of overrepresentation in repressed chromatin include vlincRNA 2375_K562 (chr8), ucaRNA X_10_345_b_hg38 (chr10), lncRNA LINC02476 (chr7). They may participate in the functioning of the PRC2 complex, similar to KCNQ1OT1.

Supplementary note S3

We reproduced the loop annotation on three datasets generated in the study on CTCF RNA-binding domain, including the wild type, and obtained a consistent number of detected loops compared to the original study (WT: 5040, ZF1d: 1269, ZF10d: 4664, examples are shown in Supplementary Figure S16). Next, we divided the loops into two groups: those that persisted between the wild-type (WT) and the deletions, and those that disappeared. For these groups we analyzed the number of interactions of the RADICL-seq protocol for mESC cells. We hypothesized that if loop formation is influenced by the CTCF RNA-binding domain, we would observe more RD-interactions with these loops than with others.

Supplementary note S4

We varied the shift value (0.5, 2, 10 megabases) and got fairly consistent results (Supplementary Figure S26). We settled on 2 megabases, since this value exceeds the size of most of the TADs (data in the literature differs greatly depending on the algorithm, data coverage and cell line: 880 Kb for mouse [3], 185 Kb for human [4]; using the TopDom[5] we got 290 Kb for K562 and 180 Kb for mESC). This allows us to assume a sufficient level of independence between the real and background models. When shifting the coordinates of the Hi-C peaks, we kept them belonging to A/B compartments, in order to preserve the plaid pattern on the Hi-C map and take into account the openness of chromatin (the importance of this is demonstrated in the results section about chromatin loops, Supplementary Figure S13).

Supplementary note S5

When setting a universal threshold for peaks q-values, the total number of peaks in different data sets differs greatly (in some cases by orders of magnitude). This can be explained by the different reads coverage and differences in the experimental procedure, which lead to different data quality, which causes differences in the binomial assessment of the significance of interactions. A similar problem occurs when analyzing ChIP-seq [6] data, where it is often solved by analyzing the reproducibility level of peaks between replicas, instead of setting a universal threshold for q-value. To smooth out these technical differences, we varied the thresholds by the q-value of peaks for different data sets, based on the assumption that the data is very noisy and the proportion of specific interactions should not be large. We have chosen a threshold of 10% of contacts falling into peaks.

References

1. Zhang, X.; Hamblin, M.H.; Yin, K.J. The long noncoding RNA Malat1: Its physiological and pathophysiological functions. *RNA Biology* **2017**, *14*, 1705–1714. https://doi.org/10.1080/15476286.2017.1358347.
2. West, J.A.; Davis, C.P.; Sunwoo, H.; Simon, M.D.; Sadreyev, R.I.; Wang, P.I.; Tolstorukov, M.Y.; Kingston, R.E. The long noncoding RNAs NEAT1 and MALAT1 bind active chromatin sites. *Molecular cell* **2014**, *55*, 791. Publisher: NIH Public Access, https://doi.org/10.1016/j.molcel.2014.07.012.
3. Dixon, J.R.; Selvaraj, S.; Yue, F.; Kim, A.; Li, Y.; Shen, Y.; Hu, M.; Liu, J.S.; Ren, B. Topological domains in mammalian genomes identified by analysis of chromatin interactions. *Nature* **2012**, *485*, 376–380. https://doi.org/10.1038/nature11082.
4. Rao, S.; Huntley, M.; Durand, N.; Stamenova, E.; Bochkov, I.; Robinson, J.; Sanborn, A.; Machol, I.; Omer, A.; Lander, E.; et al. A 3D Map of the Human Genome at Kilobase Resolution Reveals Principles of Chromatin Looping. *Cell* **2014**, *159*, 1665–1680. https://doi.org/10.1016/j.cell.2014.11.021.
5. Shin, H.; Shi, Y.; Dai, C.; Tjong, H.; Gong, K.; Alber, F.; Zhou, X.J. TopDom: an efficient and deterministic method for identifying topological domains in genomes. *Nucleic Acids Research* **2016**, *44*, e70–e70. https://doi.org/10.1093/nar/gkv1505.
6. Bailey, T.; Krajewski, P.; Ladunga, I.; Lefebvre, C.; Li, Q.; Liu, T.; Madrigal, P.; Taslim, C.; Zhang, J. Practical Guidelines for the Comprehensive Analysis of ChIP-seq Data. *PLoS Computational Biology* **2013**, *9*, e1003326. https://doi.org/10.1371/journal.pcbi.1003326.
